# Supplementary material for: Defining complicated urinary tract infection and route of antibiotics in children presenting to the emergency department: a cohort study using the Melbourne RUPERT clinical score
Source: BMJ Open. 2024 Jul 8;14(7):e082222. doi: 10.1136/bmjopen-2023-082222 (PMC11256032; doi:10.1136/bmjopen-2023-082222)
Supplement: Supplementary data [file bmjopen-2023-082222supp001.pdf]

Supplemental table 1. Sensitivity, specificity and classification accuracy of the Melbourne RUPERT score

**Cut-off**	**Sensitivity (%)**	**Specificity (%)**	**Proportion of patients correctly classified (%)**
>=0	100	0	21
>=1	100	23	40
>=2	97	48	58
>=3	77	81	80
>=4	31	95	81
>=5	14	99	81
>5	0	100	79
