# Supplementary material for: Defining complicated urinary tract infection and route of antibiotics in children presenting to the emergency department: a cohort study using the Melbourne RUPERT clinical score
Source: BMJ Open. 2024 Jul 8;14(7):e082222. doi: 10.1136/bmjopen-2023-082222 (PMC11256032; doi:10.1136/bmjopen-2023-082222)
Supplement: Supplementary data [file bmjopen-2023-082222supp002.pdf]

BMJ Publishing Group Limited (BMJ) disclaims all liability and responsibility arising from any reliance placed on this supplemental material which has been supplied by the author(s)

Supplemental table 2. Comparison of clinical features in derivation and validation cohorts

	**Derivation No. (%) n=167**	**Validation No. (%) n=168**	**p value**
Age, y (mean±SD)	5±3.1	5.1±3.2	0.94
Female	129 (77)	138 (82)	0.27
Clinical features (prior to presentation or in the ED)			
Fever	113 (68)	106 (63)	0.38
Rigors	14 (8)	18 (11)	0.47
Vomiting	59 (35)	56 (33)	0.7
Lethargy	32 (19)	33 (20)	0.91
Abdominal pain	78 (47)	74 (44)	0.63
Flank pain/tenderness	25 (15)	31 (18)	0.39
Dysfunctional voiding	6 (4)	8 (5)	0.59
Dysuria	70 (42)	63 (38)	0.41
Offensive urine	113 (68)	106 (63)	0.13
Gross haematuria	14 (8)	18 (11)	0.99
Frequency	59 (35)	56 (33)	0.18
Suprapubic tenderness	32 (19)	33 (20)	0.51
Previous history			
Urological abnormality*	37 (22)	32 (19)	0.48
Any previous documented UTI	76 (46)	86 (51)	0.3
Recurrent (≥3) documented UTI	47 (28)	47 (28)	0.97
Antibiotic prophylaxis (current)	10 (6)	8 (5)	0.62
Known resistant organism	29 (17)	28 (17)	0.86
Prior oral antibiotics this UTI	34 (20)	28 (17)	0.43
Clinical observations			
Tachycardia (any recorded in ED)	45 (27)	39 (23)	0.43
Tachycardia when afebrile	8 (5)	5 (3)	0.39

Data presented are n (%) unless stated otherwise. SD – standard deviation, ED – emergency department, UTI – Urinary tract infection, fever – ≥38°C, y – years, SD – standard deviation
