# Supplementary material for: Defining complicated urinary tract infection and route of antibiotics in children presenting to the emergency department: a cohort study using the Melbourne RUPERT clinical score
Source: BMJ Open. 2024 Jul 8;14(7):e082222. doi: 10.1136/bmjopen-2023-082222 (PMC11256032; doi:10.1136/bmjopen-2023-082222)
Supplement: Supplementary data [file bmjopen-2023-082222supp003.pdf]

Supplemental table 3: Performance of the Melbourne RUPERT score in all cohorts

	ROC AUC	Score	Sensitivity	Specificity	Proportion of patients correctly identified	Proportion of patients assigned to consider IV
Derivation and validation cohorts						
Derivation (12m-11y confirmed UTI)	0.85	3/6	77%	81%	80%	31%
		2/6	97%	48%	58%	62%
		4/6	31%	95%	81%	11%
Validation (12m-11y confirmed UTI)	0.80	3/6	67%	78%	76%	30%
Test cohorts						
12m-11y probable UTI	0.82	3/6	54%	85%	82%	18%
12y-17y all UTI	0.86	3/6	57%	86%	81%	21%
3m-11m all UTI	0.58	3/6	43%	72%	64%	32%

ROC=receiver operator curve, AUC=area under the curve, IV – intravenous, m – months, y – years, UTI – urinary tract infection
