# Supplementary material for: Defining complicated urinary tract infection and route of antibiotics in children presenting to the emergency department: a cohort study using the Melbourne RUPERT clinical score
Source: BMJ Open. 2024 Jul 8;14(7):e082222. doi: 10.1136/bmjopen-2023-082222 (PMC11256032; doi:10.1136/bmjopen-2023-082222)
Supplement: Author's manuscript [file bmjopen-2023-082222.draft_revisions.pdf]

BMJ Open is committed to open peer review. As part of this commitment we make the peer review history of every article we publish publicly available.

When an article is published we post the peer reviewers' comments and the authors' responses online. We also post the versions of the paper that were used during peer review. These are the versions that the peer review comments apply to.

The versions of the paper that follow are the versions that were submitted during the peer review process. They are not the versions of record or the final published versions. They should not be cited or distributed as the published version of this manuscript.

BMJ Open is an open access journal and the full, final, typeset and author-corrected version of record of the manuscript is available on our site with no access controls, subscription charges or pay-per-view fees (http://bmjopen.bmj.com).

If you have any questions on BMJ Open's open peer review process please email
info.bmjopen@bmj.com

BMJ Open

Defining complicated urinary tract infection and route of antibiotics in paediatric patients: a clinical score

Journal:	*BMJ Open*
Manuscript ID	bmjopen-2023-082222
Article Type:	Original research
Date Submitted by the Author:	18-Nov-2023
Complete List of Authors:	Scanlan, Barry; University of Melbourne VCCC,<br>Bryant, Penelope; The Royal Children's Hospital Melbourne, Department of General Medicine; University of Melbourne VCCC, Department of Paediatrics<br>Babl, Franz; The Royal Children's Hospital Melbourne, Emergency Department; University of Melbourne VCCC, Department of Paediatrics<br>Ibrahim, Laila; The Royal Children's Hospital Melbourne; Murdoch Children's Research Institute<br>Hopper, Sandy; The Royal Children's Hospital Melbourne, Emergency Department<br>McNab, Sarah; The Royal Children's Hospital Melbourne, General Medicine; University of Melbourne VCCC, Department of Paediatrics<br>Donath, Susan; Murdoch Children's Research Institute, Clinical Epidemiology and Biostatistics Unit<br>Davidson, Andrew; The Royal Children's Hospital Melbourne; University of Melbourne VCCC
Keywords:	Pyelonephritis < NEPHROLOGY, Urinary tract infections < UROLOGY, Paediatric A&E and ambulatory care < PAEDIATRICS, Paediatric infectious disease & immunisation < PAEDIATRICS

SCHOLARONE™
Manuscripts

I, the Submitting Author has the right to grant and does grant on behalf of all authors of the Work (as defined in the below author licence), an exclusive licence and/or a non-exclusive licence for contributions from authors who are: i) UK Crown employees; ii) where BMJ has agreed a CC-BY licence shall apply, and/or iii) in accordance with the terms applicable for US Federal Government officers or employees acting as part of their official duties; on a worldwide, perpetual, irrevocable, royalty-free basis to BMJ Publishing Group Ltd ("BMJ") its licensees and where the relevant Journal is co-owned by BMJ to the co-owners of the Journal, to publish the Work in this journal and any other BMJ products and to exploit all rights, as set out in our licence.

The Submitting Author accepts and understands that any supply made under these terms is made by BMJ to the Submitting Author unless you are acting as an employee on behalf of your employer or a postgraduate student of an affiliated institution which is paying any applicable article publishing charge ("APC") for Open Access articles. Where the Submitting Author wishes to make the Work available on an Open Access basis (and intends to pay the relevant APC), the terms of reuse of such Open Access shall be governed by a Creative Commons licence – details of these licences and which Creative Commons licence will apply to this Work are set out in our licence referred to above.

Other than as permitted in any relevant BMJ Author's Self Archiving Policies, I confirm this Work has not been accepted for publication elsewhere, is not being considered for publication elsewhere and does not duplicate material already published. I confirm all authors consent to publication of this Work and authorise the granting of this licence.

For peer review only

Defining complicated urinary tract infection and route of antibiotics in paediatric patients: a clinical score

Barry T Scanlan MB BCh BAO[a,f], Laila F Ibrahim MB BCh BAO[a,f], Franz E Babl MD[a,b,f], Sandy M Hopper FRACP[a,b,f], Sarah McNab PhD[a,c,f], Susan Donath[f], Andrew Davidson MD [a,e,f], Penelope A Bryant PhD[a,c,d,f,g]

Affiliations
a Department of Paediatrics, University of Melbourne
b Emergency Department, The Royal Children's Hospital
c Department of General Medicine, The Royal Children's Hospital
d Infectious Diseases Unit, Department of General Medicine, The Royal Children's Hospital
e Department of Anaesthesia, The Royal Children's Hospital
f Murdoch Children's Research Institute
g Hospital-In-The-Home Department, The Royal Children's Hospital
50 Flemington Road, Parkville, Victoria 3052, Australia

Corresponding author
A/Prof. Penelope A Bryant
Department of General Medicine, The Royal Children's Hospital Melbourne
50 Flemington Road, Parkville, VIC 3052, Australia
Email: penelope.bryant@rch.org.au
Tel: +613 93455522 Fax: +613 9345 6667

Key words
Clinical score, prediction rule, decision rule, urinary tract infection, complicated, pyelonephritis, acute pyelonephritis, antibiotics, intravenous, oral

Word count: 2989

For peer review only

Abstract

Objectives

Most children with urinary tract infections (UTI) can be managed with oral antibiotics. However, identifying those likely to fail oral and need intravenous (IV) antibiotics due to complications is challenging. We aimed to derive, validate and test a score to guide initial antibiotic route.

Design

This study assessed children (3 months–17 years) treated in the Emergency Department (ED) with UTI. Patients were divided into 2 groups based on IV or oral antibiotic route after 24 hours, including if they switched. Children with confirmed UTI were used to derive then validate the score, comparing complicating clinical features between the 2 groups. Combinations of significantly differentiating features generated receiver operating characteristic curves and the optimal cut-off for IV antibiotic use was selected.

Results

1,240 patients were included. 167 children aged 12m–11y with confirmed UTI comprised the derivation cohort. The combination of features that performed optimally (area under curve 0.85, 95%CI 0.79-0.91) were: rigors, urological abnormality, pyrexia (≥38°C), emesis, recurrent (>2) UTI, tachycardia: the RUPERT score (one point each, maximum 6). A score ≥3 to start IV antibiotics accurately classified 80% patients (sensitivity 77%, specificity 81%). For the 168 patients in the validation cohort, the score accurately classified 76% (sensitivity 67%, specificity 78%). The score tested well in 'probable' UTI and adolescents, and less well in infants.

Conclusion

The Melbourne RUPERT score provides the first standardised, easy-to-use score to aid clinicians in deciding route of antibiotics for complicated UTI in children. It now needs prospective validation.

For peer review only

Article Summary

Strengths and limitations of this study

The Melbourne RUPERT score:

- Is applicable to a broad range of children with UTI, offers straightforward, clinically-relevant guidance for assessing which patients with complicating features of UTI may benefit from initial IV antibiotic treatment.
- Offers a potential new approach to defining complicated UTI.
- Has been internally validated and tested on different patient cohorts.
- Has the potential limitation that in the absence of an accepted gold standard for route of treatment, we have accepted that the treating clinicians made the correct judgement after 24 hours of treatment.
- Requires testing prospectively and in other settings, particularly in infants under 12 months.

Funding statement

This study was funded by a grant from the RCH Foundation, which also in part supported BTS. PAB was supported in part by a Melbourne Campus Clinician-Scientist Fellowship, Melbourne, Australia. FEB was supported in part by a grant from the RCH Foundation and a Melbourne Campus Clinician-Scientist Fellowship, Melbourne, Australia and a National Health and Medical Research Council (NHMRC) Practitioner Fellowship, Canberra, Australia. The emergency research group, MCRI, is in part supported by an NHMRC Centre for Research Excellence Grant for Paediatric Emergency Medicine, Canberra, Australia and the Victorian government infrastructure support program.

Competing interests statement

All authors have indicated they have no financial relationships relevant to this article to disclose. The funding bodies do not have any authority in design and conduct of the study; collection, management, analysis, and interpretation of the data; and preparation, review, or approval of the manuscript.

Author Contributions

Dr Scanlan was involved in the design and co-ordinated the study, carried out the initial and subsequent data analysis, drafted the initial manuscript, revised subsequent drafts and approved the final manuscript as submitted. Drs Bryant, Babl, Davidson, Ibrahim, McNab and Hopper were involved in the concept and design of the study, provided input into data analysis, reviewed and revised the manuscript and approved the final draft. Ms Donath was involved in the design of the study, advised on statistical analysis, revised and approved the final manuscript as submitted. All authors can take responsibility for the integrity of the data and the accuracy of the data analysis. All authors approved the final manuscript.

Acknowledgments

We would like to acknowledge the participation of patients and families at RCH and clinical staff in the ED.

For peer review only

INTRODUCTION

Urinary tract infections (UTI) are common in children, with a cumulative rate up to aged 12 years of 0.7% in boys and 2.8% in girls[1]. Antibiotics are the mainstay of treatment, and most children are successfully treated with oral antibiotics. Systematic reviews have concluded that oral antibiotics are sufficient for both lower UTI[2] and uncomplicated upper UTI[3], including with fever, and these recommendations have been incorporated into American Academy of Pediatrics (AAP)[4] and UK National Institute for Health and Care Excellence (NICE) guidelines[5]. For UTI with complications, intravenous (IV) antibiotics are often – but not always – used. It is challenging to identify which children with complications should receive initial IV antibiotics because trials have variably excluded those with urological abnormality[6-9], previous UTI[6, 7], vomiting[7, 9], dehydration[7], or prior oral antibiotics[6-8]. The decision between IV and oral is important: not using IV antibiotics when they are needed risks clinical deterioration, while overuse places unnecessary burden on children and the healthcare system.

In a previous study at our hospital, 72% children presenting to the Emergency Department (ED) had at least one of these complicating features and 28% received IV antibiotics[10]. Although there is no universal definition of complicated UTI, preliminary data showed that with increased number of complicating features, there was increased likelihood of IV antibiotic use[10]. When several factors inform treatment choice, clinical scores can help by combining important features[11]. We hypothesized that combining complicating features of UTI would generate a clinical threshold to determine route of antibiotics, and could provide an approach to defining complicated UTI.

We aimed to derive, validate and test a score that combines complicating clinical features and incorporates existing evidence to guide choice of initial route of antibiotics in ED in children with UTI.

For peer review only - http://bmjopen.bmj.com/site/about/guidelines.xhtml

METHODS

Study design: A clinical cohort of consecutive patients presenting to ED with UTI was used to derive, validate and test a clinical score for defining complicated UTI and initial route of antibiotics.

Setting: The study was conducted in ED at The Royal Children's Hospital Melbourne, a tertiary paediatric hospital, from May 2016–March 2018.

Inclusion/exclusion criteria: All children aged 3 months–17 years inclusive with likely UTI in ED were eligible, and included prospectively if they fulfilled NICE diagnostic criteria[5] for symptoms and urinalysis if available (box 1). To include sufficient patients to validate the findings, participants were also identified retrospectively through diagnostic coding of UTI. Patients were excluded if they were treated in ED primarily for severe infection (e.g. sepsis or meningitis), had immunodeficiency or chronic renal failure/post renal transplant.

Clinical procedure and patient follow-up: After consent, data were collected during ED assessment, and included demographics, clinical features (prior to and in ED), previous history and urine results. Clinical observations recorded were tachycardia (heart rate >95th centile by age[12]), tachycardia when afebrile, and hypotension (systolic blood pressure <5th centile by 50th height centile[13]). The same data were collected from retrospective participants. Data were entered into REDCap (Research Electronic Data Capture[14] at Murdoch Children's Research Institute). The decision to start IV or oral antibiotics was made by an experienced ED physician (at least registrar or fellow level). Ongoing antibiotic route was re-assessed at 24 hours for patients in hospital. Patients discharged from ED on oral antibiotics were presumed to remain on oral unless they re-presented to our hospital and were switched to IV. To test the assumption they were not re-presenting to other hospitals, we contacted all prospective patients within 7

For peer review only - http://bmjopen.bmj.com/site/about/guidelines.xhtml

days, and none re-presented to other hospitals.

Standard for assessing treatment: There is no gold standard to determine which children with UTI need IV antibiotics. A standard for assessing appropriate antibiotic route was defined using existing evidence and published methodology. For a UTI clinical score to be useful, lower and upper UTI should be included as the distinction can be challenging in young children. Existing studies show children with lower UTI and upper uncomplicated UTI do not need IV antibiotics[2, 3]. For patients excluded from those studies – those with complications of vomiting, dehydration, urological abnormality, previous UTI, or oral antibiotics prior to presentation – a published method for establishing a standard for appropriate route was applied: 'the route of ongoing antibiotic treatment after review by 24 hours is likely to be appropriate based on ongoing symptom progression, even if antibiotics were administered via the other route at presentation'[15]. Therefore, if a patient on IV antibiotics was reviewed within 24 hours, and switched to oral antibiotics, they were determined to have only needed oral antibiotics initially (box 1). Likewise, if a patient treated with oral antibiotics re-presented within 24 hours and was started on IV, they were determined to have needed IV antibiotics initially. Based on this standard, patients were allocated to one of two groups: 'Oral antibiotics applicable' (no complicating features[3] *or* complicating features and on oral after 24 hours) or 'Likely to fail oral antibiotics' (complicating features and on IV after 24 hours).

Study cohorts: The derivation, validation and test cohorts were:

Derivation cohort: To determine which complicating features are important for a clinical prediction score, patients were included aged 12 m–11y with confirmed UTI – NICE diagnostic criteria plus positive urine or blood culture[16] (box 1). This age range was defined because patients with UTI under the age of 12 months are frequently managed differently[17], our previous

For peer review only - http://bmjopen.bmj.com/site/about/guidelines.xhtml

study showed large variation under 12 months[18], and studies of childhood UTI characteristically have an upper limit of 12 years[19].

Validation cohort: To assess whether the score works in a separate group defined in the same way, the 12m-11y age group with confirmed UTI was divided in half into the derivation and validation cohorts by assigning patients alternately by date of presentation. Alternate assignation avoided an external change during the study having a biased effect on one group.

Test cohorts: The score was tested on additional patient groups: 1) age 12m-11y with probable (not confirmed) UTI[16], 2) age 3m-11m, and 3) age 12y-17y.

Statistical analysis: Univariate analysis was used to compare clinical features that were different between the 'Oral antibiotics applicable' and 'Likely to fail oral antibiotics' groups. For categorical data Fisher Exact test was used and for continuous data Student t test. Odds ratios (OR) and 95% confidence intervals (95%CI) were calculated and $p<0.05$ was considered significant. Each differentiating feature between the two groups was converted to a binary score of 0 (absent) or 1 (present). Different combinations of these features were added and used to generate receiver operating characteristic (ROC) curves. Scores for each feature were weighted (increased to 2) to determine whether that improved performance. The score performance with different cut-offs for identifying those likely to fail oral antibiotics was assessed as follows: 1) high ROC area under the curve (AUC); 2) most accurate assignment of patients to the groups 'Oral antibiotics applicable' and 'Likely to fail oral antibiotics'; and 3) sensitivity and specificity. The combination of features that had the highest overall results in all of these domains was used to derive a clinical score. Statistical analysis used Stata/IC version 15.1.

Ethics: This study had ethics approval from RCH Human Research Ethics Committee (HREC36255).

For peer review only - http://bmjopen.bmj.com/site/about/guidelines.xhtml

RESULTS

Over 22 months, 1,438 patients had a diagnosis of UTI, of which 46 were excluded due to miscoding or other reasons (figure 1a). 1,392 children were treated as having a UTI in ED. Of these, 152 were excluded for not fulfilling NICE criteria (box 1) or other exclusions. Of the 1,240 with UTI, 276 (22%) were aged 3m-11m, 831 (67%) were 12m-11y and 133 (11%) were 12y-17y.

Clinical features and treatment of derivation cohort

Of 831 patients aged 12m-11y, 335 had confirmed UTI (box 1). Half (167) were assigned to the derivation cohort, and 168 to the validation cohort, with no difference between groups in triage category and hospital admission (data not shown). The treatment allocations were: derivation cohort 35 (21%) IV and 132 (79%) oral; validation cohort 47 (28%) IV and 121 (72%) oral. In the derivation cohort, there were 43 children with no complicating features and 89 with complicating features who were on oral by 24 hours (80 continuing and 9 de-escalated from IV) totalling 132 in the 'Oral antibiotics applicable' group. There were 35 children with complicating features and on IV after 24 hours (32 continuing and 3 escalated from oral) and these were assigned to the 'Likely to fail oral antibiotics' group (figure 1b).

Derivation of clinical score

Multiple patient characteristics and clinical features that have been variably included in definitions of complicated UTI or excluded from definitions of uncomplicated UTI were compared between the 'Oral antibiotics applicable' and 'Likely to fail oral antibiotics' groups (table 1). Ten features significantly differentiated between the groups: fever, rigors, vomiting, lethargy, flank pain/tenderness, dysuria, urological abnormality, any previous UTI, recurrent (>2) previous UTI, tachycardia, tachycardia when afebrile. For features that were variants of the same measure, the one that differentiated the groups more significantly was selected: i.e.

For peer review only - http://bmjopen.bmj.com/site/about/guidelines.xhtml

tachycardia differentiated more than tachycardia when afebrile, and recurrent (>2) UTI differentiated more than any previous UTI. The ROC curve of this eight-feature score had a high AUC of 0.86 (95%CI 0.81-0.92). Features were serially removed from this score and different cut-offs were tested to compare performance in terms of AUC, correct identification of 'Likely to fail oral antibiotics', sensitivity and specificity. Score performance did not deteriorate until the number of features was below six (table 3 and supplemental figure 3a).

The Melbourne RUPERT score

Using six of the eight discriminating features, the combination that performed best was rigors, urological abnormality, pyrexia, emesis, recurrent (>2) UTI and tachycardia, which can be remembered using the acronym RUPERT. Applying different weights did not affect score performance (data not shown). The score had a ROC AUC of 0.85 (95%CI 0.79-0.91) (supplemental figure 3a). Next, a RUPERT score cut-off was determined, above which patients should be considered for IV antibiotics due to having more complicated UTI, and below which oral antibiotics could be used. A cut-off of 3 – patients with a score of 3 or more considered for IV antibiotics – correctly classified 80% of patients, with sensitivity 77%, specificity 81% (table 3, figure 2). Applying a cut-off of 3, IV antibiotics would have been recommended for 52 (31%) patients and oral for 115 (69%) patients. A cut-off of 4 correctly classified 81% of patients, with sensitivity 31%, specificity 95%. With a cut-off of 4, IV antibiotics would have been recommended for 18 (11%) patients and oral for 149 (89%). Increasing the cut-off from 3 to 4 would result in increased patients failing oral treatment from 8 (5%) to 24 (14%).

Validation of the RUPERT score

The score was validated on 168 patients with the same criteria (age 12m-11y with confirmed UTI)(supplemental table 4). Applying the same standard, by 24 hours 121 (72%) were receiving oral antibiotics and 47 (28%) IV antibiotics. The RUPERT score had a similarly high

For peer review only - http://bmjopen.bmj.com/site/about/guidelines.xhtml

AUC of 0.80 (95%CI 0.73-0.88) (supplemental figure 3b) in this cohort. A score cut-off of 3 correctly classified 76% of patients (sensitivity 67%, specificity 78%). Applying a cut-off of 3, IV antibiotics would have been suggested for 51 (30%) patients oral antibiotics for 117 (70%) patients. With a cut-off of 3 in the validation cohort, 10 (6%) patients recommended for oral antibiotics would fail (table 2).

Testing the RUPERT score on other cohorts

1) *Age 12m-11y with probable UTI:* There were 498 patients in this group with a diagnosis of UTI that was likely, but not culture-confirmed[16]. Applying the score resulted in an AUC of 0.82 (95%CI 0.76-0.87), and the cut-off of 3 correctly assigned 82% of patients (sensitivity 54%, specificity 85%)(table 2)(supplemental figure 3c and supplemental table 5).
2) *Age 12y-17y with confirmed or probable UTI:* There were 133 patients in this adolescent group. Applying the score resulted in an AUC of 0.86 (95%CI 0.79-0.92), and the cut-off of 3 correctly assigned 86% of patients (sensitivity 57%, specificity 86%).
3) *Age 3m-11m with confirmed or probable UTI:* There were 276 patients in this infant group. Applying the score resulted in an AUC of only 0.58 (95%CI 0.51-0.65), and the cut-off of 3 correctly assigned 64% patients - the lowest proportion in any group (sensitivity 43%, specificity 72%).

There was no difference between the performance of the score in the 12m-17y age range between the 276 patients where data were collected prospectively (AUC 0.84, 95%CI 0.79-0.87), and the 688 patients where collection was retrospective (AUC 0.83, 95%CI 0.79-0.87).

For peer review only

DISCUSSION

The Melbourne RUPERT score is applicable to a broad range of children presenting to hospital with UTI. It offers straightforward, clinically-relevant guidance for assessing which patients with complicating features of UTI may benefit from initial IV antibiotic treatment that is currently not articulated in major guidelines. It also offers a potential new approach to defining complicated UTI: rather than a one-size-fits-all definition of specific features, it can be considered as a threshold of multiple complicating features which result in a different management pathway (IV antibiotics). Each of the features in the score is easily assessable, well-defined and objective and the score does not require patients to verbalise symptoms. Additionally, it does not rely on blood or urine results. The score performed well in pre- and primary school age children and adolescents with confirmed and probable UTI. It less accurately identified infants under 12 months receiving oral antibiotics. Although, this could be because the score does not perform well in infants, it is more likely due to the frequent initiation and/or treatment beyond 24 hours with IV antibiotics in this age group, without a clear clinical indication. There is no pathophysiological reason that infants aged 3 to 11 months should be different to older children, but both our data (45% IV initiation for all UTI in 3-11m, versus 28% in the derivation/validation cohorts) and previous studies have shown higher IV rates in this age group without clear clinical reasons[17, 18]. This therefore would benefit from prospective application and testing, and could help reduce unnecessary IV in this age group.

Using a RUPERT score cut-off of 3 provides a higher sensitivity to reduce oral antibiotic failures, at the expense of considering more patients for IV antibiotics. Alternatively, a cut-off of 4 substantially increases the number of patients recommended for oral antibiotics but reduces the sensitivity. Like most clinical scores, it performs well at the extremes and the mid-range involves a balance between sensitivity and specificity[11]. Scores in the mid-range give clinicians information about the need for monitoring in the first 24 hours of treatment.

For peer review only - http://bmjopen.bmj.com/site/about/guidelines.xhtml

The lack of a gold standard required the development of one from a combination of existing evidence (which is robust due to multiple RCTs contributing), and where evidence was limited, using published methodology derived from early clinical review. It was therefore important to ensure that clinical practice at our hospital is similar to practice elsewhere. A recent study of UTI at 36 US hospitals between 2010 and 2016 reported that in children aged 3 to 24 months, 35% were treated with IV antibiotics[20]. This is similar to 39% use in this age group at our hospital (data not shown separately).

Guidelines have proved useful in standardising management of UTI, and major international guidelines developed by NICE[5] and AAP[4] are clear in many areas of diagnosis and management (e.g. need for imaging). However, both guidelines leave treatment with IV antibiotics more flexible, suggesting that it should be based on ‘practical considerations’ (AAP) and ‘if oral antibiotics cannot be used’ (NICE). By defining patients not previously fitting under the umbrella of evidence-based recommendations, the RUPERT score adds clinical context to practical considerations and standardises when oral antibiotics cannot be used.

Incorporating a clinical score into management decisions offers an aid to reducing variation in care. Paediatric scores that have been successfully developed and implemented include the Westley score[21] for croup, the Pediatric Asthma Severity Score (PASS)[22] and the Melbourne ASSET score for cellulitis.[15] In addition to reducing variation in care, a clinical score can standardise route of antibiotics in trials, important for generalising outcomes. For example, in one study describing the outcomes of outpatient parenteral antibiotic treatment for UTI, the authors admitted that the patients could have been treated with oral antibiotics[17]. This clinical score could be used for inclusion/exclusion criteria in studies (e.g. whether participants exceed a score cut-off) and/or to describe the relative clinical severity of study subjects by recording their RUPERT score[23].

For peer review only - http://bmjopen.bmj.com/site/about/guidelines.xhtml

For peer review only

The strengths of this score, in addition to being the first to attempt to provide standardised guidance where evidence is lacking, are that it is easy to use and does not require invasive tests or waiting for culture results. In addition, by being applicable to all children with UTI because it incorporates existing strong evidence for those with uncomplicated UTI, clinicians do not need to determine whether a patient has complicated UTI before applying it. The main limitation is that it has not yet been tested prospectively or in other settings. Another potential limitation is that we have accepted that the treating clinicians in the derivation and validation cohorts made the correct judgement at 24 hours on treatment route. While these decisions are made by senior doctors, practice varies between clinicians. Review of medical records did not suggest any unusual practice, or that many patients on oral antibiotics returned. Using this score does not negate the possibility of overtreating some with IV and undertreating some with oral, but it aims to standardise the rationale for IV antibiotics, while allowing institutional flexibility to determine the treatment outcomes for which they are aiming.

For peer review only

CONCLUSION

The Melbourne RUPERT score is an easy-to-use and reproducible clinical score for UTI management in children. It incorporates robust evidence for children with uncomplicated UTI, and a new approach to defining patients with complicated UTI to standardise risk stratification and treatment recommendations, both for clinical practice and research. As with any decision-support guideline, the aim is to offer a guide rather than completely replace clinical judgement for individual patients. The score will now benefit from external validation and impact analysis.

For peer review only - http://bmjopen.bmj.com/site/about/guidelines.xhtml

References

[1] Winberg J, Andersen HJ, Bergstrom T, Jacobsson B, Larson H, Lincoln K. Epidemiology of symptomatic urinary tract infection in childhood. Acta Paediatr Scand Suppl. 1974:1-20.
[2] Fitzgerald A, Mori R, Lakhanpaul M, Tullus K. Antibiotics for treating lower urinary tract infection in children. Cochrane Database Syst Rev. 2012:CD006857.
[3] Strohmeier Y, Hodson EM, Willis NS, Webster AC, Craig JC. Antibiotics for acute pyelonephritis in children. Cochrane Database of Systematic Reviews. 2014:CD003772.
[4] Roberts KB, Downs SM, Finnell SME, Hellerstein S, Shortliffe LD, Wald ER, et al. Urinary tract infection: Clinical practice guideline for the diagnosis and management of the initial UTI in febrile infants and children 2 to 24 months. Pediatrics. 2011;128:595-610.
[5] Baumer JH, Jones RW. Urinary tract infection in children, National Institute for Health and Clinical Excellence. Arch Dis Child Educ Pract Ed. 2007;92:189-92.
[6] Hoberman A, Wald ER, Hickey RW, Baskin M, Charron M, Majd M, et al. Oral versus initial intravenous therapy for urinary tract infections in young febrile children. Pediatrics. 1999;104:79-86.
[7] Montini G, Toffolo A, Zucchetta P, Dall'Amico R, Gobber D, Calderan A, et al. Antibiotic treatment for pyelonephritis in children: Multicentre randomised controlled non-inferiority trial. British Medical Journal. 2007;335:386-8.
[8] Neuhaus TJ, Berger C, Buechner K, Parvex P, Bischoff G, Goetschel P, et al. Randomised trial of oral versus sequential intravenous/oral cephalosporins in children with pyelonephritis. European Journal of Pediatrics. 2008;167:1037-47.
[9] Bocquet N, Alaoui AS, Jais JP, Gajdos V, Guigonis V, Lacour B, et al. Randomized trial of oral versus sequential IV/Oral antibiotic for acute pyelonephritis in children. Pediatrics. 2012;129:e269-e75.
[10] Scanlan B, Ibrahim L, Hopper S, McNab S, Babl F, Davidson A, et al. 1520. Determining the Management of Children with Acute UTI/Pyelonephritis Who Do Not Fit Current Management Recommendations. Open Forum Infectious Diseases. 2018;5:S471-S.
[11] Laupacis A, Sekar N. Clinical prediction rules: a review and suggested modifications of methodological standards. Jama. 1997;277:488-94.
[12] Bonafide CP, Brady PW, Keren R, Conway PH, Marsolo K, Daymont C. Development of heart and respiratory rate percentile curves for hospitalized children. Pediatrics. 2013;131:e1150-7.
[13] Haque IU, Zaritsky AL. Analysis of the evidence for the lower limit of systolic and mean arterial pressure in children. Pediatr Crit Care Med. 2007;8:138-44.
[14] Harris PA, Taylor R, Thielke R, Payne J, Gonzalez N, Conde JG. Research electronic data capture (REDCap)--a metadata-driven methodology and workflow process for providing translational research informatics support. J Biomed Inform. 2009;42:377-81.
[15] Ibrahim LF, Hopper SM, Donath S, Salvin B, Babl FE, Bryant PA. Development and Validation of a Cellulitis Risk Score: The Melbourne ASSET Score. Pediatrics. 2019.
[16] Basmaci R, Vazouras K, Bielicki J, Folgori L, Hsia Y, Zaoutis T, et al. Urinary Tract Infection Antibiotic Trial Study Design: A Systematic Review. Pediatrics. 2017;140.
[17] Gauthier M, Chevalier I, Sterescu A, Bergeron S, Brunet S, Taddeo D. Treatment of urinary tract infections among febrile young children with daily intravenous antibiotic therapy at a day treatment center. Pediatrics. 2004;114:e469-76.
[18] Scanlan B, Ibrahim L, Quinlan C, Hopper S, Babl F, Davidson A, et al. Why are children with febrile urinary tract infection/pyelonephritis treated with intravenous antibiotics? International Paediatric Nephrology Society Association Meeting. Germany2017.

For peer review only - http://bmjopen.bmj.com/site/about/guidelines.xhtml

[19] Baker PC, Nelson DS, Schunk JE. The addition of ceftriaxone to oral therapy does not improve outcome in febrile children with urinary tract infections. Archives of Pediatrics & Adolescent Medicine. 2001;155:135-9.
[20] Chaudhari PP, Monuteaux MC, Bachur RG. Emergency Department Revisits After an Initial Parenteral Antibiotic Dose for UTI. Pediatrics. 2018;142.
[21] Westley CR, Cotton EK, Brooks JG. Nebulized racemic epinephrine by IPPB for the treatment of croup: a double-blind study. Am J Dis Child. 1978;132:484-7.
[22] Gorelick MH, Stevens MW, Schultz TR, Scribano PV. Performance of a novel clinical score, the Pediatric Asthma Severity Score (PASS), in the evaluation of acute asthma. Acad Emerg Med. 2004;11:10-8.
[23] clinical.trials.gov. Single Dose Intravenous Antibiotics for Complicated Urinary Tract Infections in Children (CHOICE UTI). 2021; 2021.
[24] Urinary tract infectionin under 16s: diagnosis and management, clinical guideline CG54.: United Kingdom National Institute for Health and Care Excellence; 2017.

For peer review only

For peer review only - http://bmjopen.bmj.com/site/about/guidelines.xhtml

Box 1: Definitions of groups

1) Definitions for eligibility

NICE clinical criteria* for diagnosis of UTI at presentation to ED

Symptoms	Age <2 years: fever, rigor, vomiting, flank pain/tenderness, poor feeding, irritability, offensive urine, gross haematuria or failure to thrive<br>Age ≥2 years: fever, rigor, vomiting, abdominal pain, flank pain/tenderness, dysfunctional voiding, change in urinary continence, dysuria, gross haematuria, frequency, offensive urine or cloudy urine
AND (if available)	
Urinalysis	Abnormal urinary dipstick leucocyte esterase >1+ or nitrite positive<br>OR<br>White cell count (WCC) >10 x10^6/l in uncentrifuged urine<br>OR<br>Bacteriuria

2) Definitions for derivation/validation/test cohorts

Confirmed UTI†

Patients who met NICE clinical criteria for diagnosis and who were treated as having UTI

AND

Culture	Positive urine culture with no more than 2 species of microorganisms >10^7 CFU/L<br>OR<br>Positive blood culture and no other recognised cause

Probable UTI

Patients who met NICE clinical criteria for diagnosis and who were treated as having UTI but NOT fulfilling research criteria recommendations for culture-based 'confirmed' UTI.

3) Definitions for group classification

'Likely to fail oral antibiotics' – complicating features (vomiting, dehydration, urological abnormality, previous UTI, or oral antibiotics prior to presentation) *and* on IV after 24 hours

'Oral antibiotics applicable' – no complicating features, or complicating features and on oral after 24 hours

UTI – urinary tract infection, ED – emergency department, CFU – colony forming units, *NICE guidelines[24] †Trial design recommendations[24]

For peer review only - http://bmjopen.bmj.com/site/about/guidelines.xhtml

Figure legend

Figure 1. Patient assignment to a) derivation, validation and test cohorts and b) within the derivation cohort to ‘Oral antibiotics applicable’ and ‘Likely to fail oral antibiotics’ groups in the derivation cohort; UTI – urinary tract infections, ED – emergency department, IV – intravenous, y – year(s), m – month(s), h – hour(s)

Figure 2. Sensitivity and specificity at each cut-off threshold of the Melbourne RUPERT score for the derivation cohort

Supplemental Figure 3. Comparison of receiver operating characteristic curves with a) different numbers of features for the derivation cohort; b) for derivation and validation cohorts; and c) for further test cohorts; (AUC (95%CI))

For peer review only

For peer review only - http://bmjopen.bmj.com/site/about/guidelines.xhtml

Table 1: Comparison between the 'Oral antibiotics applicable' and 'Likely to fail oral antibiotics' groups in the derivation cohort

	**Oral antibiotics applicable No. (%) n=132**	**Likely to fail oral antibiotics No. (%) n=35**	**OR (95%CI)**	**p value**
Age, y (mean±SD)	5.1±3.1	4.9±3.2		0.98
Female	103 (78)	26 (74)	0.8 (0.3-1.9)	0.64
Clinical features (prior to presentation or in the ED)				
Fever	81 (61)	32 (91)	6.7 (2.1-22)	<0.001
Rigors	8 (6)	6 (17)	3.2 (1.1-10)	0.04
Vomiting	41 (31)	18 (51)	2.4 (1.1-5)	0.03
Lethargy	19 (14)	13 (37)	3.5 (1.5-8)	0.002
Abdominal pain	61 (46)	17 (49)	1.1 (0.5-2.3)	0.8
Flank pain/tenderness	16 (12)	9 (26)	2.5 (1-6.2)	0.045
Dysfunctional voiding	5 (4)	1 (3)	0.8 (0-5.1)	0.79
Dysuria	62 (47)	8 (23)	0.3 (0.1-0.8)	0.01
Offensive urine	17 (13)	3 (9)	0.6 (0.2-2.1)	0.49
Gross haematuria	14 (11)	1 (3)	0.2 (0-1.5)	0.15
Frequency	23 (17)	2 (6)	0.3 (0-1.2)	0.08
Suprapubic tenderness	22 (17)	3 (9)	0.5 (0.1-1.6)	0.23
Previous history				
Urological abnormality	20 (15)	17 (49)	5.3 (2.4-11.9)	<0.001
Any previous UTI	52 (39)	24 (69)	3.4 (1.5-7.3)	0.002
Recurrent (>2) previous UTI	26 (20)	21 (60)	6.1 (2.8-13.5)	<0.001
Antibiotic prophylaxis	6 (5)	4 (11)	2.7 (0.8-9.6)	0.13
Known resistant organisms	19 (14)	10 (29)	2.4 (1-5.7)	0.05
Prior oral antibiotics	26 (20)	8 (23)	1.2 (0.5-2.9)	0.68
Clinical observations				
Tachycardia	27 (20)	18 (51)	4.1 (1.9-9)	<0.001
Tachycardia when afebrile	4 (3)	4 (3)	4.1 (1.1-16)	0.04
Hypotension	0 (0)	0 (0)	NA	NA

OR=odds ratio, SD=standard deviation, ED=emergency department, UTI=urinary tract infection, fever=≥38◦C. *prior to presentation or in the emergency department

For peer review only - http://bmjopen.bmj.com/site/about/guidelines.xhtml

Table 2: Effect on patient management to all cohorts aged 3 months-17 years

**Cohort**	**Derivation 12m-11y Confirmed UTI No. (%)**	**Validation 12m-11y Confirmed UTI No. (%)**	**Test 12m-11y Probable UTI No. (%)**	**Test 12y-17y All UTI No. (%)**	**Test 3m-11m All UTI No. (%)**	**Final 12m-17y All UTI No. (%)**
Patients	167	168	498	133	276	966
Route of initial antibiotic treatment						
Oral	124 (74)	117 (70)	421 (85)	91 (68)	153 (55)	753 (78)
IV	43 (26)	51 (30)	77 (15)	42 (32)	123 (45)	213 (22)
Route of antibiotic treatment by 24 hours						
Oral	132 (79)	138 (82)	448 (90)	112 (84)	197 (71)	830 (86)
IV	35 (21)	30 (18)	50 (10)	21 (16)	79 (29)	136 (14)
Melbourne RUPERT score assignation						
Recommend oral (score <3)	115 (69)	117 (70)	406 (82)	105 (79)	188 (68)	743 (77)
Recommend IV (score ≥3)	52 (31)	51 (30)	92 (18)	28 (21)	88 (32)	223 (23)

UTI – urinary tract infection, IV – intravenous

For peer review only

Table 3. Sensitivity, specificity and classification accuracy of the Melbourne RUPERT score

Cut-off	Sensitivity (%)	Specificity (%)	Proportion of patients correctly classified (%)
>=0	100	0	21
>=1	100	23	40
>=2	97	48	58
>=3	77	81	80
>=4	31	95	81
>=5	14	99	81
>5	0	100	79

For peer review only

For peer review only - http://bmjopen.bmj.com/site/about/guidelines.xhtml

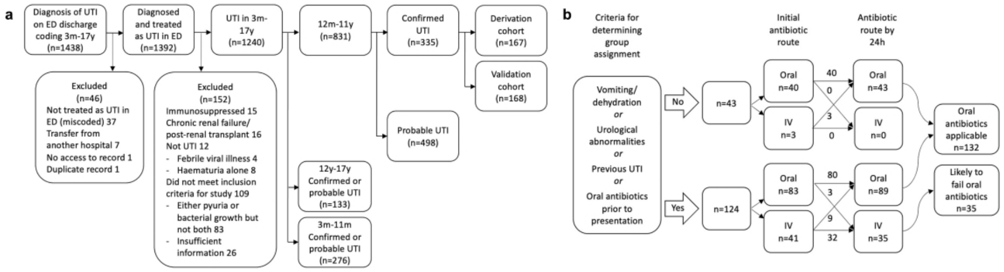

Patient assignment to a) derivation, validation and test cohorts and b) within the derivation cohort to 'Oral antibiotics applicable' and 'Likely to fail oral antibiotics' groups in the derivation cohort; UTI – urinary tract infections, ED – emergency department, IV – intravenous, y – year(s), m – month(s), h – hour(s)

284x75mm (300 x 300 DPI)

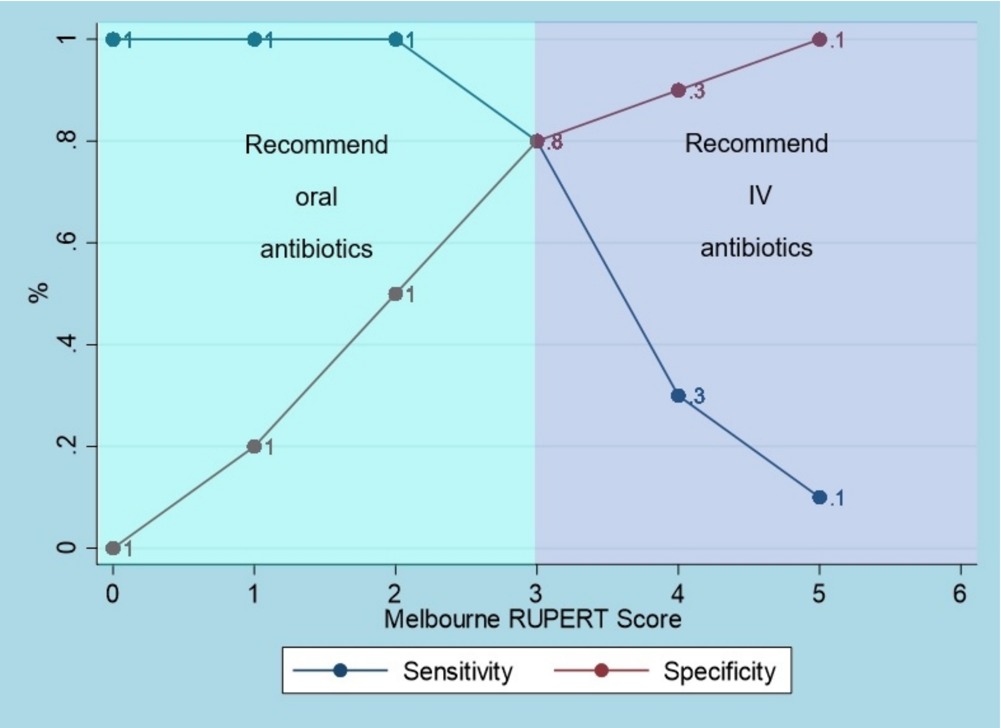

Sensitivity and specificity at each cut-off threshold of the Melbourne RUPERT score for the derivation cohort

138x100mm (144 x 144 DPI)

Supplemental table 4. Comparison of clinical features in derivation and validation cohorts

	Derivation No. (%) n=167	Validation No. (%) n=168	p value
Age, y (mean±SD)	5±3.1	5.1±3.2	0.94
Female	129 (77)	138 (82)	0.27
Clinical features (prior to presentation or in the ED)			
Fever	113 (68)	106 (63)	0.38
Rigors	14 (8)	18 (11)	0.47
Vomiting	59 (35)	56 (33)	0.7
Lethargy	32 (19)	33 (20)	0.91
Abdominal pain	78 (47)	74 (44)	0.63
Flank pain/tenderness	25 (15)	31 (18)	0.39
Dysfunctional voiding	6 (4)	8 (5)	0.59
Dysuria	70 (42)	63 (38)	0.41
Offensive urine	113 (68)	106 (63)	0.13
Gross haematuria	14 (8)	18 (11)	0.99
Frequency	59 (35)	56 (33)	0.18
Suprapubic tenderness	32 (19)	33 (20)	0.51
Previous history			
Urological abnormality	37 (22)	32 (19)	0.48
Any previous UTI	76 (46)	86 (51)	0.3
>2 Previous UTI	47 (28)	47 (28)	0.97
Antibiotic prophylaxis	10 (6)	8 (5)	0.62
Known resistant organism	29 (17)	28 (17)	0.86
Prior oral antibiotics	34 (20)	28 (17)	0.43
Clinical observations			
Tachycardia	45 (27)	39 (23)	0.43
Tachycardia when afebrile	8 (5)	5 (3)	0.39

Data presented are n (%) unless stated otherwise. SD – standard deviation, ED – emergency department, UTI – Urinary tract infection, fever – ≥38°C, y – years, SD – standard deviation

For peer review only

Supplemental table 5: Performance of the Melbourne RUPERT score in all cohorts

	ROC AUC	Score	Sensitivity	Specificity	Proportion of patients correctly identified	Proportion of patients assigned to consider IV
Derivation and validation cohorts						
Derivation	0.85	3/6	77%	81%	80%	31%
(12m-11y		2/6	97%	48%	58%	62%
confirmed UTI)		4/6	31%	95%	81%	11%
Validation (12m-11y confirmed UTI)	0.80	3/6	67%	78%	76%	30%
Test cohorts						
12m-11y probable UTI	0.82	3/6	54%	85%	82%	18%
12y-17y all UTI	0.86	3/6	57%	86%	81%	21%
3m-11m all UTI	0.58	3/6	43%	72%	64%	32%

ROC=receiver operator curve, AUC=area under the curve, IV – intravenous, m – months, y – years, UTI – urinary tract infection

For peer review only

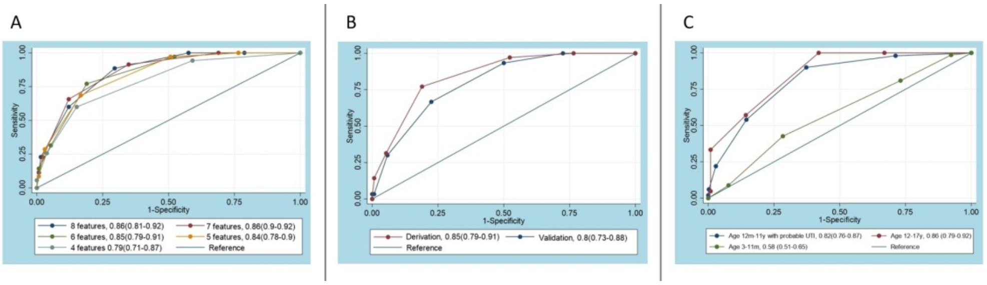

380x105mm (120 x 120 DPI)

Success criteria for Clinical Decision Rules[1] – check list completed for the Melbourne RUPERT score

For a Clinical Decision Rule to be considered successful, all of the following should apply:

General

- *Need:* Outlined in the introduction

Rule Derivation Methodology

- *Setting and Population:* This rule was derived using a representative population, children attending the ED, for which it is intended to be used.
- *Outcome:* As described is both clinically important and consistently assessed
- *Predictor Variables:* The predictive variables used were reliably recorded and are clinically relevant. These were based on patient history and observed vital signs in the ED, this produced a clinical score with features that are easily assessable, well-defined and objective and the score does not require the patient to verbalise symptoms. Additionally, this score does not rely on results of blood or urine tests. The score was shown to be equally reliable when the information was collected prospectively and retrospectively. Observers inputted variables prior to the creation of the score and were therefore unaware of which variables would be used.
- *Rule creation:* The rule was created incorporating best available evidence and following previously published methodology as described.

Rule Derivation Reporting

- *Performance:* Analysis including receiver operating characteristic (ROC) curves, sensitivity and specificity are clearly and prominently reported both in the text and in figures, 95% confidence intervals are reported.
- *Success:* The score offers an aid to clinical decision making as was the aim, it is easy-to-use and reproducible. While the score has been internally validated it would benefit from external validation and impact analysis as stated.
- *Reliability:* The rule performed reliably in those patients aged 12 months to 17 years old.

Reference:

1. Green SM, Schriger DL, Yealy DM. Methodologic standards for interpreting clinical decision rules in emergency medicine: 2014 update. *Ann Emerg Med.* 2014;64(3):286-291.

For peer review only

Study Protocol

Study design: A clinical cohort of consecutive patients presenting to ED with UTI was used to derive, validate and test a clinical score for defining complicated UTI and initial route of antibiotics.

Setting: The study will be conducted in ED at The Royal Children's Hospital Melbourne, a tertiary paediatric hospital, from May 2016.

Inclusion/exclusion criteria: All children aged 3 months–17 years inclusive with likely UTI in ED are eligible, and included prospectively if they fulfilled NICE diagnostic criteria[5] for symptoms and urinalysis if available. To include sufficient patients to validate the findings, participants will also be identified retrospectively through diagnostic coding of UTI. Patients will be excluded if they are treated in ED primarily for severe infection (e.g. sepsis or meningitis), have immunodeficiency or chronic renal failure/post renal transplant.

Clinical procedure and patient follow-up: After consent, data will be collected during ED assessment, and included demographics, clinical features (prior to and in ED), previous history and urine results. Clinical observations recorded will be tachycardia (heart rate >95th centile by age[12]), tachycardia when afebrile, and hypotension (systolic blood pressure <5th centile by 50th height centile[13]). The same data will be collected from retrospective participants. Data will be entered into REDCap (Research Electronic Data Capture[14] at Murdoch Children's Research Institute). The decision to start IV or oral antibiotics will be made by an experienced ED physician (at least registrar or fellow level). Ongoing antibiotic route will be re-assessed at 24 hours for patients in hospital. Patients discharged from ED on oral antibiotics will be presumed to remain on oral unless they re-presented to our hospital and are switched to IV. To test the assumption they are not re-presenting to other hospitals, we will contact all prospective patients within 7 days, and ask if they have re-presented to other hospitals.

Standard for assessing treatment: There is no gold standard to determine which children with UTI need IV antibiotics. A standard for assessing appropriate antibiotic route was defined using existing evidence and published methodology. For a UTI clinical score to be useful, lower and upper UTI should be included as the distinction can be challenging in young children. Existing studies show children with lower UTI and upper uncomplicated UTI do not need IV antibiotics[2, 3]. For patients excluded from those studies – those with complications of vomiting, dehydration, urological abnormality, previous UTI, or oral antibiotics prior to presentation – a published method for establishing a standard for appropriate route was applied: 'the route of ongoing antibiotic treatment after review by 24 hours is likely to be appropriate based on ongoing symptom progression, even if antibiotics were administered via the other route at presentation'[15]. Therefore, if a patient on IV antibiotics was reviewed within 24 hours, and switched to oral antibiotics, they will be determined to have only needed oral antibiotics initially. Likewise, if a patient treated with oral antibiotics re-presented within 24 hours and was started on IV, they will be determined to have needed IV antibiotics initially. Based on this standard, patients were allocated to one of two groups: 'Oral antibiotics applicable' (no complicating features[3] *or* complicating features and on oral after 24 hours) or 'Likely to fail oral antibiotics' (complicating features and on IV after 24 hours).

Study cohorts: The derivation, validation and test cohorts will be:
Derivation cohort: To determine which complicating features are important for a clinical prediction score, patients were included aged 12 m–11y with confirmed UTI – NICE diagnostic criteria plus

For peer review only - http://bmjopen.bmj.com/site/about/guidelines.xhtml

positive urine or blood culture[16] . This age range was defined because patients with UTI under the age of 12 months are frequently managed differently[17], our previous study showed large variation under 12 months[18], and studies of childhood UTI characteristically have an upper limit of 12 years[19].
Validation cohort: To assess whether the score works in a separate group defined in the same way, the 12m-11y age group with confirmed UTI will be divided in half into the derivation and validation cohorts by assigning patients alternately by date of presentation. Alternate assignation avoided an external change during the study having a biased effect on one group.
Test cohorts: The score will be tested on additional patient groups: 1) age 12m-11y with probable (not confirmed) UTI[16], 2) age 3m-11m, and 3) age 12y-17y.

Statistical analysis: Univariate analysis will be used to compare clinical features that are different between the 'Oral antibiotics applicable' and 'Likely to fail oral antibiotics' groups. For categorical data Fisher Exact test will be used and for continuous data Student t test. Odds ratios (OR) and 95% confidence intervals (95%CI) will be calculated and $p<0.05$ will be considered significant. Each differentiating feature between the two groups will be converted to a binary score of 0 (absent) or 1 (present). Different combinations of these features will be added and used to generate receiver operating characteristic (ROC) curves. Scores for each feature will be weighted (increased to 2) to determine whether that improved performance. The score performance with different cut-offs for identifying those likely to fail oral antibiotics will be assessed as follows: 1) high ROC area under the curve (AUC); 2) most accurate assignment of patients to the groups 'Oral antibiotics applicable' and 'Likely to fail oral antibiotics'; and 3) sensitivity and specificity. The combination of features that has the highest overall results in all of these domains was used to derive a clinical score. Statistical analysis will use Stata/IC.

Ethics: This study has ethics approval from RCH Human Research Ethics Committee (HREC36255).

For peer review only

BMJ Open

Defining complicated urinary tract infection and route of antibiotics in paediatric patients: the RUPERT clinical score

Journal:	*BMJ Open*
Manuscript ID	bmjopen-2023-082222.R1
Article Type:	Original research
Date Submitted by the Author:	07-Mar-2024
Complete List of Authors:	Scanlan, Barry; University of Melbourne VCCC,<br>Bryant, Penelope; The Royal Children's Hospital Melbourne, Department of General Medicine; University of Melbourne VCCC, Department of Paediatrics<br>Babl, Franz; The Royal Children's Hospital Melbourne, Emergency Department; University of Melbourne VCCC, Department of Paediatrics<br>Ibrahim, Laila; The Royal Children's Hospital Melbourne; Murdoch Children's Research Institute<br>Hopper, Sandy; The Royal Children's Hospital Melbourne, Emergency Department<br>McNab, Sarah; The Royal Children's Hospital Melbourne, General Medicine; University of Melbourne VCCC, Department of Paediatrics<br>Donath, Susan; Murdoch Children's Research Institute, Clinical Epidemiology and Biostatistics Unit<br>Davidson, Andrew; The Royal Children's Hospital Melbourne; University of Melbourne VCCC
**Primary Subject Heading**:	Paediatrics
Secondary Subject Heading:	Emergency medicine, Infectious diseases
Keywords:	Pyelonephritis < NEPHROLOGY, Urinary tract infections < UROLOGY, Paediatric A&E and ambulatory care < PAEDIATRICS, Paediatric infectious disease & immunisation < PAEDIATRICS

SCHOLARONE™
Manuscripts

I, the Submitting Author has the right to grant and does grant on behalf of all authors of the Work (as defined in the below author licence), an exclusive licence and/or a non-exclusive licence for contributions from authors who are: i) UK Crown employees; ii) where BMJ has agreed a CC-BY licence shall apply, and/or iii) in accordance with the terms applicable for US Federal Government officers or employees acting as part of their official duties; on a worldwide, perpetual, irrevocable, royalty-free basis to BMJ Publishing Group Ltd ("BMJ") its licensees and where the relevant Journal is co-owned by BMJ to the co-owners of the Journal, to publish the Work in this journal and any other BMJ products and to exploit all rights, as set out in our licence.

The Submitting Author accepts and understands that any supply made under these terms is made by BMJ to the Submitting Author unless you are acting as an employee on behalf of your employer or a postgraduate student of an affiliated institution which is paying any applicable article publishing charge ("APC") for Open Access articles. Where the Submitting Author wishes to make the Work available on an Open Access basis (and intends to pay the relevant APC), the terms of reuse of such Open Access shall be governed by a Creative Commons licence – details of these licences and which Creative Commons licence will apply to this Work are set out in our licence referred to above.

Other than as permitted in any relevant BMJ Author's Self Archiving Policies, I confirm this Work has not been accepted for publication elsewhere, is not being considered for publication elsewhere and does not duplicate material already published. I confirm all authors consent to publication of this Work and authorise the granting of this licence.

For peer review only

FDefining complicated urinary tract infection and route of antibiotics in paediatric patients: the RUPERT clinical score

Barry T Scanlan MB BCh BAO[a,f], Laila F Ibrahim MB BCh BAO[a,f], Franz E Babl MD[a,b,f], Sandy M Hopper FRACP[a,b,f], Sarah McNab PhD[a,c,f], Susan Donath[f], Andrew Davidson MD [a,e,f], Penelope A Bryant PhD[a,c,d,f,g]

Affiliations
a Department of Paediatrics, University of Melbourne
b Emergency Department, The Royal Children's Hospital
c Department of General Medicine, The Royal Children's Hospital
d Infectious Diseases Unit, Department of General Medicine, The Royal Children's Hospital
e Department of Anaesthesia, The Royal Children's Hospital
f Murdoch Children's Research Institute
g Hospital-In-The-Home Department, The Royal Children's Hospital
50 Flemington Road, Parkville, Victoria 3052, Australia

Corresponding author
A/Prof. Penelope A Bryant
Department of General Medicine, The Royal Children's Hospital Melbourne
50 Flemington Road, Parkville, VIC 3052, Australia
Email: penelope.bryant@rch.org.au
Tel: +613 93455522 Fax: +613 9345 6667

Key words
urinary tract infection, pyelonephritis, paediatric A&E and ambulatory care, paediatric infectious disease and immunisation

Word count: 2979 - revised manuscript 3420

For peer review only
For peer review only - http://bmjopen.bmj.com/site/about/guidelines.xhtml

Abstract

Objectives

Most children with uncomplicated urinary tract infections (UTI) can be managed with oral antibiotics. However, identifying those likely to fail oral and need intravenous (IV) antibiotics due to complicating features at presentation is challenging. We aimed to derive, validate and test a score to guide initial antibiotic route.

Design

This study assessed children (3 months–17 years) treated in the Emergency Department (ED) with UTI, enrolled both prospectively and retrospectively. Patients were divided into 2 groups based on IV or oral antibiotic route after 24 hours, including if they switched. Children with confirmed UTI were used to derive then validate the score, comparing complicating clinical features between the 2 groups. Combinations of significantly differentiating features generated receiver operating characteristic curves and the optimal cut-off for IV antibiotic use was selected.

Results

A total of 1,240 patients were included. 167 children aged 12m–11y with confirmed UTI comprised the derivation cohort. The combination of features that performed optimally (area under curve 0.85, 95%CI 0.79-0.91) were: rigors, urological abnormality, pyrexia (≥38°C), emesis, recurrent (≥3) UTI, tachycardia: the RUPERT score (one point each, maximum 6). A score ≥3 accurately classified route of antibiotics after 24 hours for 80% patients (sensitivity 77%, specificity 81%). For the 168 patients in the validation cohort, the score accurately classified 76% (sensitivity 67%, specificity 78%). The score tested well in 'probable' UTI and adolescents, and less well in infants.

Conclusion

The Melbourne RUPERT score provides the first standardised, easy-to-use score to aid clinicians in deciding route of antibiotics for more complicated UTI in children. It now needs prospective validation.

For peer review only - http://bmjopen.bmj.com/site/about/guidelines.xhtml

Article Summary

Strengths and limitations of this study

The Melbourne RUPERT score:

- Is applicable to a broad range of children with UTI, offers straightforward, clinically-relevant guidance for assessing which patients with complicating features of UTI may benefit from initial IV antibiotic treatment.
- Offers a potential new approach to defining complicated UTI.
- Has been internally validated and tested on different patient cohorts.
- Has the potential limitation that in the absence of an accepted gold standard for route of treatment, we have accepted that the treating clinicians made the correct judgement after 24 hours of treatment.
- Requires testing prospectively and in other settings, particularly in infants under 12 months.

Funding statement

This study was funded by a grant from the RCH Foundation (RCHF 2016-718 [CM68607]), which also in part supported BTS. PAB was supported in part by a Melbourne Campus Clinician-Scientist Fellowship, Melbourne, Australia. FEB was supported in part by a grant from the RCH Foundation and a Melbourne Campus Clinician-Scientist Fellowship, Melbourne, Australia and a National Health and Medical Research Council (NHMRC) Practitioner Fellowship, Canberra, Australia. The emergency research group, MCRI, is in part supported by an NHMRC Centre for Research Excellence Grant for Paediatric Emergency Medicine, Canberra, Australia and the Victorian government infrastructure support program.

Competing interests statement

All authors have indicated they have no financial relationships relevant to this article to disclose. The funding bodies do not have any authority in design and conduct of the study; collection, management, analysis, and interpretation of the data; and preparation, review, or approval of the manuscript.

Data sharing

No additional data available. Ethics committee approval can be sought to share data on request.

Author Contributions

Dr Scanlan was involved in the design and co-ordinated the study, carried out the initial and subsequent data analysis, drafted the initial manuscript, revised subsequent drafts and approved the final manuscript as submitted. A/Prof Bryant, Prof Babl, Prof Davidson, Dr Ibrahim, Dr McNab and A/Prof Hopper were involved in the concept and design of the study, provided input into data analysis, reviewed and revised the manuscript and approved the final draft. A/Prof Donath was involved in the design of the study, advised on statistical analysis, revised and approved the final manuscript as submitted. All authors take responsibility for the integrity of the data and the accuracy of the data analysis. All authors approved the final manuscript.

Acknowledgments

We would like to acknowledge the participation of patients and families at RCH and

For peer review only

clinical staff in the ED.

For peer review only

For peer review only - http://bmjopen.bmj.com/site/about/guidelines.xhtml

INTRODUCTION

Urinary tract infections (UTI) are common in children, with a prevalence rate in by the age 7 years of 1-2% in boys and 7-8% in girls[1 2]. Antibiotics are the mainstay of treatment, and most children are successfully treated with oral antibiotics. The decision between IV and oral is important: not using IV antibiotics when they are needed risks clinical deterioration, while overuse places unnecessary burden on children and the healthcare system. Systematic reviews have concluded that oral antibiotics are sufficient for both lower UTI[3] and uncomplicated upper UTI[4], including with fever, and these recommendations have been incorporated into American Academy of Pediatrics (AAP)[5] and UK National Institute for Health and Care Excellence (NICE) guidelines[6]. For complicated UTI, intravenous (IV) antibiotics are often – but not always – used. There is no consensus on the definition of complicated UTI: definitions variably include multiple factors related to past history and current presentation that may result in divergence from typical management. It is challenging to identify which children with a variety of complicating features at presentation should receive initial IV antibiotics because trials have variably excluded those with urological abnormality[7-10], previous UTI[7 8], vomiting[8 10], dehydration[8], or prior oral antibiotics[7-9], so recommendations are less robust.

In a previous study at our hospital, 72% children presenting to the Emergency Department (ED) had at least one of these complicating features and 28% received IV antibiotics[11]. Although there is no universal definition of complicated UTI, preliminary data showed that with increased number of complicating features, there was increased likelihood of initial IV antibiotic use[11]. When several factors inform treatment choice, clinical scores can help by combining important features[12]. We hypothesized that combining complicating features of UTI would generate a clinical threshold to determine route of antibiotics, and could provide an approach to defining complicated UTI.

We aimed to derive, validate and test a score that combines complicating clinical features and

For peer review only - http://bmjopen.bmj.com/site/about/guidelines.xhtml

incorporates existing evidence to guide choice of initial route of antibiotics in ED in children with UTI.

METHODS

Study design: A clinical population of consecutive patients presenting to ED with UTI was used to derive, validate and test a clinical score for defining complicated UTI and initial route of antibiotics.

Setting: The study was conducted in ED at The Royal Children's Hospital Melbourne, a tertiary paediatric hospital, from May 2016–March 2018.

Inclusion/exclusion criteria: All children aged 3 months–17 years inclusive with likely UTI in ED were eligible, and included if they fulfilled NICE diagnostic criteria[6] for symptoms and urinalysis (box 1). Patients were enrolled both prospectively to ensure integrity of the information collected, and also retrospectively to include sufficient patients to validate the findings. Patients were excluded if they were treated in ED via superseding pathways: severe infection (e.g. sepsis or meningitis) or immunodeficiency (e.g. febrile neutropenia or post renal transplant).

Patient and Public Involvement: No patient involved.

Clinical procedure and patient follow-up: After consent, data were collected using a standardised case record form during ED assessment for patients enrolled prospectively, and included demographics, clinical features prior to and in ED (table 1), previous history including urological abnormality (defined in box 1) and urine results. Clinical observations recorded were fever ≥38°C, tachycardia (heart rate >95th centile by age[13]), tachycardia when afebrile, and

For peer review only - http://bmjopen.bmj.com/site/about/guidelines.xhtml

hypotension (systolic blood pressure <5th centile by 50th height centile[14]). Prospective data collected were compared with the electronic medical record and on determining a high degree of concordance, the same data were collected from retrospective participants using the same criteria. The concordance enabled missing clinical features in the retrospective clinical record review to be presumed absent. Data were entered into REDCap (Research Electronic Data Capture[15] at Murdoch Children's Research Institute). The decision to start IV or oral antibiotics was made by an experienced ED physician (at least registrar or fellow level). Institutional empiric guidelines for IV antibiotics for UTI recommend gentamicin 24 hourly and benzylpenicillin 6 hourly. Ongoing antibiotic route was re-assessed at 24 hours for patients in hospital. Patients discharged from ED on oral antibiotics were presumed to remain on oral unless they re-presented to our hospital and were switched to IV. To test the assumption they were not re-presenting to other hospitals, we contacted all prospective patients within 7 days, and none re-presented to other hospitals.

Standard for assessing treatment: There is no gold standard to determine which children with UTI need IV antibiotics. A standard for assessing appropriate antibiotic route was defined using existing evidence and published methodology. For a UTI clinical score to be useful, lower and upper UTI should be included as the distinction can be challenging in young children. Existing studies show children with lower UTI and upper uncomplicated UTI do not need IV antibiotics[3 4]. For patients excluded from those studies – those with complications of vomiting, dehydration, urological abnormality, previous UTI, or oral antibiotics prior to presentation – a published method for establishing a standard for appropriate route was applied: 'the route of ongoing antibiotic treatment after review by 24 hours is likely to be appropriate based on ongoing symptom progression, even if antibiotics were administered via the other route at presentation'[16]. Therefore, if a patient on IV antibiotics was reviewed within 24 hours, and switched to oral antibiotics, they were determined to have only needed oral antibiotics initially

For peer review only - http://bmjopen.bmj.com/site/about/guidelines.xhtml

(box 1). Likewise, if a patient treated with oral antibiotics re-presented within 24 hours and was started on IV, they were determined to have needed IV antibiotics initially. Based on this standard, patients were allocated to one of two groups: ‘Oral antibiotics applicable’ (no complicating features[4] *or* complicating features and on oral after 24 hours) or ‘Likely to fail oral antibiotics’ (complicating features and on IV after 24 hours).

Study cohorts: The derivation, validation and test cohorts were:

Derivation cohort: To determine which complicating features are important for a clinical prediction score, patients were included aged 12 m–11y with confirmed UTI – NICE diagnostic criteria plus positive urine or blood culture[17] (box 1). This age range was defined because patients with UTI under the age of 12 months are frequently managed differently[18], our previous study showed large variation under 12 months[19], and studies of childhood UTI characteristically have an upper limit of 12 years[20].

Validation cohort: To assess whether the score works in a separate group defined in the same way, the 12m-11y age group with confirmed UTI was divided in half into the derivation and validation cohorts by assigning patients alternately by date of presentation. Alternate assignation avoided an external change during the study having a biased effect on one group.

Test cohorts: The score was tested on additional patient groups: 1) age 12m-11y with probable (not confirmed) UTI[17], 2) age 3m-11m, and 3) age 12y-17y.

Statistical analysis: Univariate analysis was used to compare clinical features that were different between the ‘Oral antibiotics applicable’ and ‘Likely to fail oral antibiotics’ groups. For categorical data Fisher Exact test was used and for continuous data Student t test. Odds ratios (OR) and 95% confidence intervals (95%CI) were calculated and $p<0.05$ was considered significant. Each differentiating feature between the two groups was converted to a binary score of 0 (absent) or 1 (present). Different combinations of these features were added and used to

For peer review only

generate receiver operating characteristic (ROC) curves. Scores for each feature were weighted (increased to 2) to determine whether that improved performance. The score performance with different cut-offs for identifying those likely to fail oral antibiotics was assessed as follows: 1) high ROC area under the curve (AUC); 2) most accurate assignment of patients to the groups 'Oral antibiotics applicable' and 'Likely to fail oral antibiotics'; and 3) sensitivity and specificity. The combination of features that had the highest overall results in all of these domains was used to derive a clinical score. Statistical analysis used Stata/IC version 15.1.

Ethics: This study had ethics approval from RCH Human Research Ethics Committee (HREC36255).

RESULTS

Over 22 months, 1,438 patients had a diagnosis of UTI, of which 46 were excluded due to miscoding or other reasons (figure 1a). 1,392 children were treated as having a UTI in ED. Of these, 152 were excluded for not fulfilling NICE criteria (box 1) or other exclusions, resulting in 1,240 patients with UTI. Of these 1,240, 276 (22%) were aged 3m-11m, 831 (67%) were 12m-11y and 133 (11%) were 12y-17y.

Clinical features and treatment of derivation cohort

Of 831 patients aged 12m-11y, 335 had confirmed UTI (box 1). Half (167) were assigned to the derivation cohort, and 168 to the validation cohort, with no difference between groups in triage category and hospital admission (data not shown). The treatment allocations at 24 hours were: derivation cohort 35 (21%) IV and 132 (79%) oral; validation cohort 47 (28%) IV and 121 (72%) oral. In the derivation cohort, there were 43 children with no complicating features and 89 with complicating features who were on oral by 24 hours (80 continuing and 9 de-escalated from IV) totalling 132 in the 'Oral antibiotics applicable' group. Of the 9 who de-escalated, 8 had only

For peer review only - http://bmjopen.bmj.com/site/about/guidelines.xhtml

For peer review only

a 24-hourly dosed antibiotic, and 1 additionally had multi-dose antibiotics per day. There were 35 children with complicating features and on IV after 24 hours (32 continuing and 3 escalated from oral) and these were assigned to the 'Likely to fail oral antibiotics' group (figure 1b).

Derivation of clinical score

Multiple patient characteristics and clinical features that have been variably included in definitions of complicated UTI or excluded from definitions of uncomplicated UTI were compared between the 'Oral antibiotics applicable' and 'Likely to fail oral antibiotics' groups. Ten features significantly differentiated between the groups (either recorded in ED or reported in the previous 24 hours): fever ≥38°C, rigors, vomiting, lethargy, flank pain/tenderness, dysuria, urological abnormality (known anatomical or functional anomaly of any part of the urological tract), previous UTI, documented recurrent (≥3) UTI, any tachycardia, tachycardia when afebrile (table 1). For features that were variants of the same measure, the one that differentiated the groups more significantly was selected: 'any tachycardia' differentiated more than 'tachycardia when afebrile', and 'recurrent (≥3) UTI' differentiated more than 'any previous UTI'. The ROC curve of this eight-feature score had a high AUC of 0.86 (95%CI 0.81-0.92). Features were serially removed from this score and different cut-offs were tested to compare performance in terms of AUC, correct identification of 'Likely to fail oral antibiotics', sensitivity and specificity. Score performance did not deteriorate until the number of features was below six (figure 2a). There was expected overlap between some features: for example, in the total population, 217 (18%) children had both fever and tachycardia, while 92 (7%) had tachycardia when afebrile and 444 (36%) had fever without tachycardia. Likewise, 130 (6%) had both urological abnormality and recurrent UTI, while 214 (10%) patients had urological abnormality without recurrent UTI and 261 (12%) patients had recurrent UTI without documented abnormality.

For peer review only - http://bmjopen.bmj.com/site/about/guidelines.xhtml

The Melbourne RUPERT score

Using six of the eight discriminating features, the combination that performed best was rigors, urological abnormality, pyrexia, emesis, recurrent (>2) UTI and tachycardia, which can be remembered using the acronym RUPERT. Applying different weights did not affect score performance (data not shown). The score had a ROC AUC of 0.85 (95%CI 0.79-0.91) (figure 2a). Next, a RUPERT score cut-off was determined, above which patients should be considered for IV antibiotics due to having more complicated UTI, and below which oral antibiotics could be used. A cut-off of 3 – patients with a score of 3 or more considered for IV antibiotics – correctly classified the route of antibiotics after 24 hours for 80% of patients, with sensitivity 77%, specificity 81% (table 2 , figure 3). Applying a cut-off of 3, IV antibiotics would have been recommended for 52 (31%) patients and oral for 115 (69%) patients. This threshold for use of IV may act as a surrogate for identifying more complicated UTI. A cut-off of 4 correctly classified 81% of patients, with sensitivity 31%, specificity 95%. With a cut-off of 4, IV antibiotics would have been recommended for 18 (11%) patients and oral for 149 (89%). Increasing the cut-off from 3 to 4 would result in increased patients failing oral treatment from 8 (5%) to 24 (14%). For the 9 who de-escalated from IV to oral by 24 hours, 2 had a score of 1 (both recurrent UTI), 4 scored 2 (2 fever and vomiting, 1 fever and tachycardia, 1 fever and urological abnormality), 1 scored 3 (fever, rigors and vomiting), and 2 scored 4 (both fever, vomiting, recurrent UTI and urological abnormality – 1 with neurogenic bladder, 1 with pyeloplasty).

Validation of the RUPERT score

The score was validated on 168 patients with the same criteria (age 12m-11y with confirmed UTI)(supplemental table 1). Applying the same standard, by 24 hours 121 (72%) were receiving oral antibiotics and 47 (28%) IV antibiotics. The RUPERT score had a similarly high AUC of 0.80 (95%CI 0.73-0.88) (figure 2b) in this cohort. A score cut-off of 3 correctly classified 76%

For peer review only - http://bmjopen.bmj.com/site/about/guidelines.xhtml

For peer review only

of patients (sensitivity 67%, specificity 78%). Applying a cut-off of 3, IV antibiotics would have been suggested for 51 (30%) patients oral antibiotics for 117 (70%) patients. With a cut-off of 3 in the validation cohort, 10 (6%) patients recommended for oral antibiotics would fail (supplemental table 2).

Testing the RUPERT score on other cohorts

1) *Age 12m-11y with probable UTI:* There were 498 patients in this group with a diagnosis of UTI that was likely, but not culture-confirmed[17]. Applying the score resulted in an AUC of 0.82 (95%CI 0.76-0.87), and the cut-off of 3 correctly assigned 82% of patients (sensitivity 54%, specificity 85%)(supplemental table 1)(figure 2c and supplemental table 3).
2) *Age 12y-17y with confirmed or probable UTI:* There were 133 patients in this adolescent group. Applying the score resulted in an AUC of 0.86 (95%CI 0.79-0.92), and the cut-off of 3 correctly assigned 86% of patients (sensitivity 57%, specificity 86%).
3) *Age 3m-11m with confirmed or probable UTI:* There were 276 patients in this infant group. Applying the score resulted in an AUC of only 0.58 (95%CI 0.51-0.65), and the cut-off of 3 correctly assigned 64% patients - the lowest proportion in any group (sensitivity 43%, specificity 72%).

There was no difference between the performance of the score in the 12m-17y age range between the 276 patients where data were collected prospectively (AUC 0.84, 95%CI 0.79-0.87), and the 688 patients where collection was retrospective (AUC 0.83, 95%CI 0.79-0.87).

DISCUSSION

The Melbourne RUPERT score is applicable to a broad range of children presenting to hospital with UTI. It offers straightforward, clinically-relevant guidance for assessing which patients with complicating features of UTI may benefit from initial IV antibiotic treatment that is currently not

For peer review only - http://bmjopen.bmj.com/site/about/guidelines.xhtml

articulated in major guidelines. It also offers a potential new approach to defining complicated UTI: rather than a one-size-fits-all definition of specific features, it can be considered as a threshold of multiple complicating features which result in a different management pathway (IV antibiotics). Each of the features in the score is easily assessable, well-defined and objective and the score does not require patients to verbalise symptoms. Additionally, it does not rely on invasive blood tests or waiting for urine culture results. The score performed well in pre- and primary school age children and adolescents with confirmed and probable UTI. It less accurately identified infants under 12 months receiving oral antibiotics. Although, this could be because the score does not perform well in infants, it is more likely due to the frequent initiation and/or treatment beyond 24 hours with IV antibiotics in this age group, without a clear clinical indication. There is no pathophysiological reason that infants aged 3 to 11 months should be different to older children, but both our data (45% IV initiation for all UTI in 3-11m, versus 28% in the derivation/validation cohorts) and previous studies have shown higher IV rates in this age group without clear clinical reasons[18 19]. This therefore would benefit from prospective application and testing, and could help reduce unnecessary IV in this age group.

Using a RUPERT score cut-off of 3 provides a higher sensitivity to reduce oral antibiotic failures, at the expense of considering more patients for IV antibiotics. Alternatively, a cut-off of 4 substantially increases the number of patients recommended for oral antibiotics but reduces the sensitivity. Like most clinical scores, it performs well at the extremes and the mid-range involves a balance between sensitivity and specificity[12]. Scores in the mid-range give clinicians information about the need for monitoring in the first 24 hours of treatment. Scores in the lower range reassure that even with features potentially associated with sepsis (rigors, tachycardia), in isolation do not necessitate IV antibiotic use. We did not seek to exclude features that were more likely to occur together (e.g. fever and tachycardia, urological abnormality and recurrent UTI), as

For peer review only - http://bmjopen.bmj.com/site/about/guidelines.xhtml

co-occurrence may signal increased severity of illness or risk of rapid damage to the kidney, when the IV antibiotics would be appropriate.

The lack of a gold standard required the development of one from a combination of existing evidence (which is robust due to multiple RCTs contributing), and where evidence was limited, using published methodology derived from early clinical review. It was therefore important to ensure that clinical practice at our hospital is similar to practice elsewhere. A recent study of UTI at 36 US hospitals between 2010 and 2016 reported that in children aged 3 to 24 months, 35% were treated with IV antibiotics[21]. This is similar to 39% use in this age group at our hospital (data not shown separately).

Guidelines have proved useful in standardising management of UTI, and major international guidelines developed by NICE[6] and AAP[5] are clear in many areas of diagnosis and management (e.g. need for imaging). However, both guidelines leave treatment with IV antibiotics more flexible, suggesting that it should be based on 'practical considerations' (AAP) and 'if oral antibiotics cannot be used' (NICE). By defining patients not previously fitting under the umbrella of evidence-based recommendations, the RUPERT score adds clinical context to practical considerations and standardises recommendations for when oral antibiotics are more likely to fail. It allows for an individualised approach by using an individual child's own clinical features rather than trying to impose a particular definition of complicated UTI, which is additionally lacking evidence for management .

Incorporating a clinical score into management decisions offers an aid to reducing variation in care. Paediatric scores that have been successfully developed and implemented include the Westley score[22] for croup, the Pediatric Asthma Severity Score (PASS)[23] and the Melbourne ASSET score for cellulitis.[16] In addition to reducing variation in care, a clinical

For peer review only - http://bmjopen.bmj.com/site/about/guidelines.xhtml

score can standardise route of antibiotics in trials, important for generalising outcomes. For example, in one study describing the outcomes of outpatient parenteral antibiotic treatment for UTI, the authors admitted that the patients could have been treated with oral antibiotics[18]. This clinical score could be used for inclusion/exclusion criteria in studies (e.g. whether participants exceed a score cut-off) and/or to describe the relative clinical severity of study subjects by recording their RUPERT score[24].

The strengths of this score, in addition to being the first to attempt to provide standardised guidance where evidence is lacking, are that it is easy to use and does not require invasive tests or waiting for culture results. In addition, by being applicable to all children with UTI because it incorporates existing strong evidence for those with uncomplicated UTI, clinicians do not need to determine whether a patient has complicated UTI before applying it. The main limitation is that it has not yet been tested prospectively or in other settings. Another potential limitation is that we have accepted that the treating clinicians in the derivation and validation cohorts made the correct judgement at 24 hours on treatment route. While these decisions are made by senior doctors, practice varies between clinicians. Review of medical records did not suggest any unusual practice, or that many patients on oral antibiotics returned. Using this score does not negate the possibility of overtreating some with IV and undertreating some with oral, but it aims to standardise the rationale for IV antibiotics, while allowing institutional flexibility to determine the treatment outcomes for which they are aiming.

CONCLUSION

The Melbourne RUPERT score is an easy-to-use and reproducible clinical score for UTI management in children. It incorporates robust evidence for children with uncomplicated UTI, and a new approach to defining patients with complicated UTI to standardise risk stratification and treatment recommendations, both for clinical practice and research. As with any decision-

For peer review only - http://bmjopen.bmj.com/site/about/guidelines.xhtml

support tool, the aim is to offer a guide rather than completely replace clinical judgement for individual patients. The score will now benefit from external validation and impact analysis.

For peer review only

For peer review only - http://bmjopen.bmj.com/site/about/guidelines.xhtml

References

1. Shaikh N, Morone NE, Bost JE, et al. Prevalence of urinary tract infection in childhood: A meta-analysis. *Pediatric Infectious Disease Journal* 2008;27(4):302-08. doi: 10.1097/INF.0b013e31815e4122
2. Sood A, Penna FJ, Eleswarapu S, et al. Incidence, admission rates, and economic burden of pediatric emergency department visits for urinary tract infection: data from the nationwide emergency department sample, 2006 to 2011. *Journal of Pediatric Urology* 2015;11(5):246. e1-46. e8.
3. Fitzgerald A, Mori R, Lakhanpaul M, et al. Antibiotics for treating lower urinary tract infection in children. *Cochrane Database of Systematic Reviews* 2012(8):CD006857. doi: 10.1002/14651858.CD006857.pub2 [published Online First: 2012/08/17]
4. Strohmeier Y, Hodson EM, Willis NS, et al. Antibiotics for acute pyelonephritis in children. *Cochrane Database of Systematic Reviews* 2014(7):CD003772. doi: https://dx.doi.org/10.1002/14651858.CD003772.pub4
5. Roberts KB, Downs SM, Finnell SME, et al. Urinary tract infection: Clinical practice guideline for the diagnosis and management of the initial UTI in febrile infants and children 2 to 24 months. *Pediatrics* 2011;128(3):595-610. doi: 10.1542/peds.2011-1330
6. Baumer JH, Jones RW. Urinary tract infection in children, National Institute for Health and Clinical Excellence. *Archives of Disease in Childhood: Education and Practice Edition* 2007;92(6):189-92. doi: 10.1136/adc.2007.130799
7. Hoberman A, Wald ER, Hickey RW, et al. Oral versus initial intravenous therapy for urinary tract infections in young febrile children. *Pediatrics* 1999;104(1 Pt 1):79-86.
8. Montini G, Toffolo A, Zucchetta P, et al. Antibiotic treatment for pyelonephritis in children: Multicentre randomised controlled non-inferiority trial. *British Medical Journal* 2007;335(7616):386-88. doi: 10.1136/bmj.39244.692442.55
9. Neuhaus TJ, Berger C, Buechner K, et al. Randomised trial of oral versus sequential intravenous/oral cephalosporins in children with pyelonephritis. *European Journal of Pediatrics* 2008;167(9):1037-47. doi: https://dx.doi.org/10.1007/s00431-007-0638-1
10. Bocquet N, Alaoui AS, Jais JP, et al. Randomized trial of oral versus sequential IV/Oral antibiotic for acute pyelonephritis in children. *Pediatrics* 2012;129(2):e269-e75. doi: http://dx.doi.org/10.1542/peds.2011-0814
11. Scanlan B, Ibrahim L, Hopper S, et al. 1520. Determining the Management of Children with Acute UTI/Pyelonephritis Who Do Not Fit Current Management Recommendations. *Open Forum Infectious Diseases* 2018;5(suppl_1):S471-S71. doi: 10.1093/ofid/ofy210.1349
12. Laupacis A, Sekar N. Clinical prediction rules: a review and suggested modifications of methodological standards. *Journal of the American Medical Association* 1997;277(6):488-94.
13. Bonafide CP, Brady PW, Keren R, et al. Development of heart and respiratory rate percentile curves for hospitalized children. *Pediatrics* 2013;131(4):e1150-7. doi: 10.1542/peds.2012-2443 [published Online First: 2013/03/13]
14. Haque IU, Zaritsky AL. Analysis of the evidence for the lower limit of systolic and mean arterial pressure in children. *Pediatric Critical Care Medicine* 2007;8(2):138-44. doi: 10.1097/01.PCC.0000257039.32593.DC [published Online First: 2007/02/03]
15. Harris PA, Taylor R, Thielke R, et al. Research electronic data capture (REDCap)--a metadata-driven methodology and workflow process for providing translational research informatics support. *Journal of Biomedical Informatics* 2009;42(2):377-81. doi: 10.1016/j.jbi.2008.08.010 [published Online First: 2008/10/22]

For peer review only

16. Ibrahim LF, Hopper SM, Donath S, et al. Development and Validation of a Cellulitis Risk Score: The Melbourne ASSET Score. *Pediatrics* 2019 doi: 10.1542/peds.2018-1420 [published Online First: 2019/01/05]
17. Basmaci R, Vazouras K, Bielicki J, et al. Urinary Tract Infection Antibiotic Trial Study Design: A Systematic Review. *Pediatrics* 2017;140(6) doi: 10.1542/peds.2017-2209 [published Online First: 2017/12/01]
18. Gauthier M, Chevalier I, Sterescu A, et al. Treatment of urinary tract infections among febrile young children with daily intravenous antibiotic therapy at a day treatment center. *Pediatrics* 2004;114(4):e469-76.
19. Scanlan B, Ibrahim L, Quinlan C, et al. Why are children with febrile urinary tract infection/pyelonephritis treated with intravenous antibiotics? International Paediatric Nephrology Society Association Meeting. Germany, 2017.
20. Baker PC, Nelson DS, Schunk JE. The addition of ceftriaxone to oral therapy does not improve outcome in febrile children with urinary tract infections. *Archives of Pediatrics & Adolescent Medicine* 2001;155(2):135-9.
21. Chaudhari PP, Monuteaux MC, Bachur RG. Emergency Department Revisits After an Initial Parenteral Antibiotic Dose for UTI. *Pediatrics* 2018;142(3) doi: 10.1542/peds.2018-0900 [published Online First: 2018/08/23]
22. Westley CR, Cotton EK, Brooks JG. Nebulized racemic epinephrine by IPPB for the treatment of croup: a double-blind study. *The American Journal of Diseases of Children* 1978;132(5):484-7. [published Online First: 1978/05/01]
23. Gorelick MH, Stevens MW, Schultz TR, et al. Performance of a novel clinical score, the Pediatric Asthma Severity Score (PASS), in the evaluation of acute asthma. *Academic Emergency Medicine* 2004;11(1):10-8. [published Online First: 2004/01/08]
24. clinical.trials.gov. Single Dose Intravenous Antibiotics for Complicated Urinary Tract Infections in Children (CHOICE UTI): 2021; 2021 [Available from: https://clinicaltrials.gov/ct2/show/NCT04876131?recrs=a&cond=uti&age=0&draw=3&rank=11] accessed 15/05/2023 2023.
25. Urinary tract infectionin under 16s: diagnosis and management, clinical guideline CG54.: United Kingdom National Institute for Health and Care Excellence; 2017 [Available from: https://www.nice.org.uk/Guidance/CG54 accessed 21.06.2018 2018.

For peer review only

For peer review only - http://bmjopen.bmj.com/site/about/guidelines.xhtml

Box 1: Definitions of groups

1) Definitions for eligibility
NICE clinical criteria* for diagnosis of UTI at presentation to ED

Symptoms	Age <2 years: fever, rigor, vomiting, flank tenderness, poor feeding, irritability, offensive urine, gross haematuria or failure to thrive<br>Age ≥2 years: fever, rigor, vomiting, abdominal pain, flank pain/tenderness, dysfunctional voiding, change in urinary continence, dysuria, gross haematuria, frequency, offensive urine or cloudy urine
AND (collected by clean catch, mid-stream urine, in/out catheter or supra-pubic aspirate)	
Urinalysis	Abnormal urinary dipstick leucocyte esterase >1+ or nitrite positive<br>OR<br>White cell count (WCC) >10 x10^6/l in uncentrifuged urine<br>OR<br>Bacteriuria

opinion

2) Definitions for derivation/validation/test cohorts
Confirmed UTI†
Patients who met NICE clinical criteria for diagnosis and who were treated as having UTI
AND

Culture	Positive urine culture with no more than 2 species of microorganisms >10^7 CFU/L<br>OR<br>Positive blood culture and no other recognised cause

Probable UTI
Patients who met NICE clinical criteria for diagnosis and who were treated as having UTI but NOT fulfilling research criteria recommendations for culture-based 'confirmed' UTI.

3) Definitions for group classification
'Likely to fail oral antibiotics' – complicating features (vomiting, dehydration, urological abnormality*, previous UTI, or oral antibiotics prior to presentation) *and* on IV after 24 hours

'Oral antibiotics applicable' – no complicating features, or complicating features and on oral after 24 hours

*Urological abnormalities included any known anatomical or functional abnormality of the urological tract, as deemed relevant by the physician in the management of the current UTI. These ranged from moderate/severe (e.g. ureteric obstruction, duplex kidneys with vesico-ureteric reflux (VUR), grades III-V VUR, grades III-IV hydronephrosis, neurogenic bladder, post pyeloplasty and post ureteric implantation), to mild (e.g. post hypospadias repair, grade I-II VUR, grade I-II hydronephrosis, phimosis with balanitis).

UTI – urinary tract infection, ED – emergency department, CFU – colony forming units, *NICE guidelines[25] †Trial design recommendations[25]

For peer review only - http://bmjopen.bmj.com/site/about/guidelines.xhtml

Figure legend

Figure 1. Patient assignment to a) derivation, validation and test cohorts and b) within the derivation cohort to 'Oral antibiotics applicable' and 'Likely to fail oral antibiotics' groups in the derivation cohort; UTI – urinary tract infections, ED – emergency department, IV – intravenous, y – year(s), m – month(s), h – hour(s)

Figure 2. Comparison of receiver operating characteristic curves with a) different numbers of features for the derivation cohort; b) for derivation and validation cohorts; and c) for further test cohorts; (AUC (95%CI))

Figure 3. Sensitivity and specificity at each cut-off threshold of the Melbourne RUPERT score for the derivation cohort

For peer review only

For peer review only - http://bmjopen.bmj.com/site/about/guidelines.xhtml

Table 1: Comparison between the 'Oral antibiotics applicable' and 'Likely to fail oral antibiotics' groups in the derivation cohort audit

	Oral antibiotics applicable No. (%) n=132	Likely to fail oral antibiotics No. (%) n=35	OR (95%CI)	p value
Age, y (mean±SD)	5.1±3.1	4.9±3.2		0.98
Female	103 (78)	26 (74)	0.8 (0.3-1.9)	0.64
Clinical features (either recorded in ED or reported in the previous 24 hours)				
Fever ≥38°C	81 (61)	32 (91)	6.7 (2.1-22)	<0.001
Rigors	8 (6)	6 (17)	3.2 (1.1-10)	0.04
Vomiting	41 (31)	18 (51)	2.4 (1.1-5)	0.03
Lethargy	19 (14)	13 (37)	3.5 (1.5-8)	0.002
Abdominal pain	61 (46)	17 (49)	1.1 (0.5-2.3)	0.8
Flank pain/tenderness	16 (12)	9 (26)	2.5 (1-6.2)	0.045
Dysfunctional voiding	5 (4)	1 (3)	0.8 (0-5.1)	0.79
Dysuria	62 (47)	8 (23)	0.3 (0.1-0.8)	0.01
Offensive urine	17 (13)	3 (9)	0.6 (0.2-2.1)	0.49
Gross haematuria	14 (11)	1 (3)	0.2 (0-1.5)	0.15
Frequency	23 (17)	2 (6)	0.3 (0-1.2)	0.08
Suprapubic tenderness	22 (17)	3 (9)	0.5 (0.1-1.6)	0.23
Previous history				
Urological abnormality*	20 (15)	17 (49)	5.3 (2.4-11.9)	<0.001
Any previous documented UTI	52 (39)	24 (69)	3.4 (1.5-7.3)	0.002
Recurrent (≥3) documented UTI	26 (20)	21 (60)	6.1 (2.8-13.5)	<0.001
Antibiotic prophylaxis (current)	6 (5)	4 (11)	2.7 (0.8-9.6)	0.13
Known resistant organisms†	19 (14)	10 (29)	2.4 (1-5.7)	0.05
Prior oral antibiotics this UTI	26 (20)	8 (23)	1.2 (0.5-2.9)	0.68
Clinical observations				
Tachycardia (any)	27 (20)	18 (51)	4.1 (1.9-9)	<0.001
Tachycardia when afebrile	4 (3)	4 (3)	4.1 (1.1-16)	0.04
Hypotension	0 (0)	0 (0)	NA	NA

OR: odds ratio, SD: standard deviation, ED: emergency department, UTI: urinary tract infection

*Urological abnormality: known anatomical or functional abnormality of the urological tract; this included any abnormality that the treating physician felt was relevant to the UTI management

†Resistant to the recommended antibiotics in the institutional guideline

For peer review only - http://bmjopen.bmj.com/site/about/guidelines.xhtml

For peer review only

Table 2. Sensitivity, specificity and classification accuracy of the Melbourne RUPERT score

Cut-off	Sensitivity (%)	Specificity (%)	Proportion of patients correctly classified (%)
>=0	100	0	21
>=1	100	23	40
>=2	97	48	58
>=3	77	81	80
>=4	31	95	81
>=5	14	99	81
>5	0	100	79

For peer review only

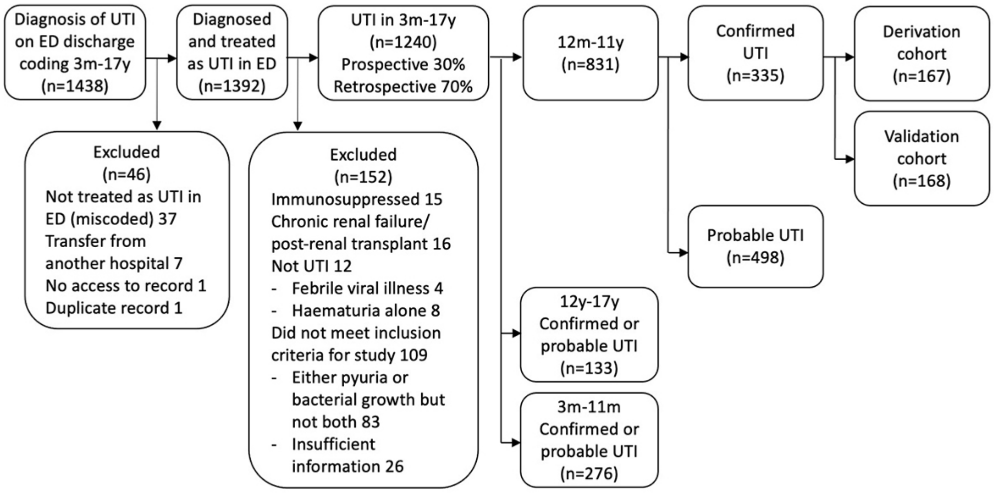

Patient assignment to a) derivation, validation and test cohorts and b) within the derivation cohort to 'Oral antibiotics applicable' and 'Likely to fail oral antibiotics' groups in the derivation cohort; UTI – urinary tract infections, ED – emergency department, IV – intravenous, y – year(s), m – month(s), h – hour(s)

149x74mm (200 x 200 DPI)

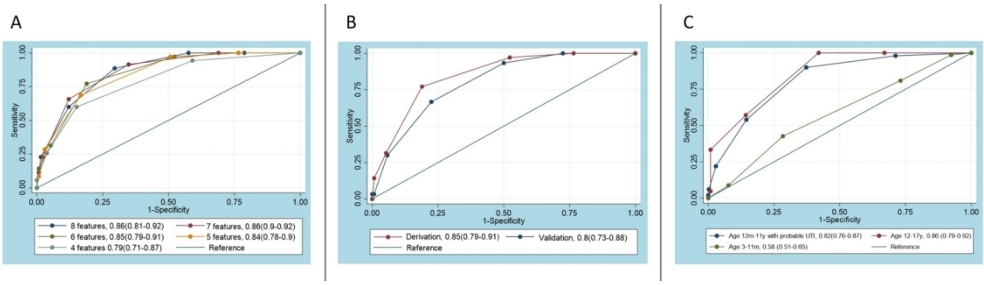

Comparison of receiver operating characteristic curves with a) different numbers of features for the derivation cohort; b) for derivation and validation cohorts; and c) for further test cohorts; (AUC (95%CI))

380x105mm (120 x 120 DPI)

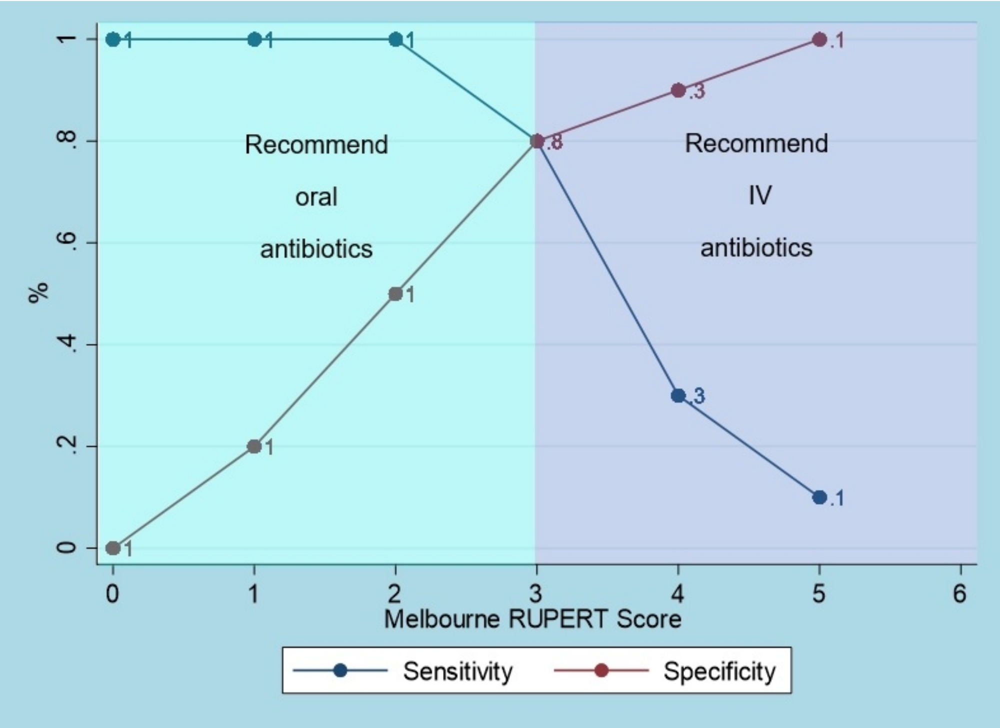

Sensitivity and specificity at each cut-off threshold of the Melbourne RUPERT score for the derivation cohort

138x100mm (144 x 144 DPI)

Supplemental table 1. Comparison of clinical features in derivation and validation cohorts

	Derivation No. (%) n=167	Validation No. (%) n=168	p value
Age, y (mean±SD)	5±3.1	5.1±3.2	0.94
Female	129 (77)	138 (82)	0.27
Clinical features (prior to presentation or in the ED)			
Fever	113 (68)	106 (63)	0.38
Rigors	14 (8)	18 (11)	0.47
Vomiting	59 (35)	56 (33)	0.7
Lethargy	32 (19)	33 (20)	0.91
Abdominal pain	78 (47)	74 (44)	0.63
Flank pain/tenderness	25 (15)	31 (18)	0.39
Dysfunctional voiding	6 (4)	8 (5)	0.59
Dysuria	70 (42)	63 (38)	0.41
Offensive urine	113 (68)	106 (63)	0.13
Gross haematuria	14 (8)	18 (11)	0.99
Frequency	59 (35)	56 (33)	0.18
Suprapubic tenderness	32 (19)	33 (20)	0.51
Previous history			
Urological abnormality	37 (22)	32 (19)	0.48
Any previous UTI	76 (46)	86 (51)	0.3
>2 Previous UTI	47 (28)	47 (28)	0.97
Antibiotic prophylaxis	10 (6)	8 (5)	0.62
Known resistant organism	29 (17)	28 (17)	0.86
Prior oral antibiotics	34 (20)	28 (17)	0.43
Clinical observations			
Tachycardia	45 (27)	39 (23)	0.43
Tachycardia when afebrile	8 (5)	5 (3)	0.39

Data presented are n (%) unless stated otherwise. SD – standard deviation, ED – emergency department, UTI – Urinary tract infection, fever – ≥38◦C, y – years, SD – standard deviation

For peer review only

Supplemental table 2. Comparison of clinical features in derivation and validation cohorts

	Derivation No. (%) n=167	Validation No. (%) n=168	p value
Age, y (mean±SD)	5±3.1	5.1±3.2	0.94
Female	129 (77)	138 (82)	0.27
Clinical features (prior to presentation or in the ED)			
Fever	113 (68)	106 (63)	0.38
Rigors	14 (8)	18 (11)	0.47
Vomiting	59 (35)	56 (33)	0.7
Lethargy	32 (19)	33 (20)	0.91
Abdominal pain	78 (47)	74 (44)	0.63
Flank pain/tenderness	25 (15)	31 (18)	0.39
Dysfunctional voiding	6 (4)	8 (5)	0.59
Dysuria	70 (42)	63 (38)	0.41
Offensive urine	113 (68)	106 (63)	0.13
Gross haematuria	14 (8)	18 (11)	0.99
Frequency	59 (35)	56 (33)	0.18
Suprapubic tenderness	32 (19)	33 (20)	0.51
Previous history			
Urological abnormality	37 (22)	32 (19)	0.48
Any previous UTI	76 (46)	86 (51)	0.3
>2 Previous UTI	47 (28)	47 (28)	0.97
Antibiotic prophylaxis	10 (6)	8 (5)	0.62
Known resistant organism	29 (17)	28 (17)	0.86
Prior oral antibiotics	34 (20)	28 (17)	0.43
Clinical observations			
Tachycardia	45 (27)	39 (23)	0.43
Tachycardia when afebrile	8 (5)	5 (3)	0.39

Data presented are n (%) unless stated otherwise. SD – standard deviation, ED – emergency department, UTI – Urinary tract infection, fever – ≥38◦C, y – years, SD – standard deviation

For peer review only

Supplemental table 3: Performance of the Melbourne RUPERT score in all cohorts

	ROC AUC	Score	Sensitivity	Specificity	Proportion of patients correctly identified	Proportion of patients assigned to consider IV
Derivation and validation cohorts						
Derivation (12m-11y confirmed UTI)	0.85	3/6	77%	81%	80%	31%
		2/6	97%	48%	58%	62%
		4/6	31%	95%	81%	11%
Validation (12m-11y confirmed UTI)	0.80	3/6	67%	78%	76%	30%
Test cohorts						
12m-11y probable UTI	0.82	3/6	54%	85%	82%	18%
12y-17y all UTI	0.86	3/6	57%	86%	81%	21%
3m-11m all UTI	0.58	3/6	43%	72%	64%	32%

ROC=receiver operator curve, AUC=area under the curve, IV – intravenous, m – months, y – years, UTI – urinary tract infection

For peer review only

Success criteria for Clinical Decision Rules[1] – check list completed for the Melbourne RUPERT score

For a Clinical Decision Rule to be considered successful, all of the following should apply:

General

- *Need:* Outlined in the introduction

Rule Derivation Methodology

- *Setting and Population:* This rule was derived using a representative population, children attending the ED, for which it is intended to be used.
- *Outcome:* As described is both clinically important and consistently assessed
- *Predictor Variables:* The predictive variables used were reliably recorded and are clinically relevant. These were based on patient history and observed vital signs in the ED, this produced a clinical score with features that are easily assessable, well-defined and objective and the score does not require the patient to verbalise symptoms. Additionally, this score does not rely on results of blood or urine tests. The score was shown to be equally reliable when the information was collected prospectively and retrospectively. Observers inputted variables prior to the creation of the score and were therefore unaware of which variables would be used.
- *Rule creation:* The rule was created incorporating best available evidence and following previously published methodology as described.

Rule Derivation Reporting

- *Performance:* Analysis including receiver operating characteristic (ROC) curves, sensitivity and specificity are clearly and prominently reported both in the text and in figures, 95% confidence intervals are reported.
- *Success:* The score offers an aid to clinical decision making as was the aim, it is easy-to-use and reproducible. While the score has been internally validated it would benefit from external validation and impact analysis as stated.
- *Reliability:* The rule performed reliably in those patients aged 12 months to 17 years old.

Reference:

1. Green SM, Schriger DL, Yealy DM. Methodologic standards for interpreting clinical decision rules in emergency medicine: 2014 update. *Ann Emerg Med.* 2014;64(3):286-291.

For peer review only

BMJ Open

Defining complicated urinary tract infection and route of antibiotics in children attending Emergency: the Melbourne RUPERT clinical score – a cohort study

Journal:	*BMJ Open*
Manuscript ID	bmjopen-2023-082222.R2
Article Type:	Original research
Date Submitted by the Author:	06-May-2024
Complete List of Authors:	Scanlan, Barry; University of Melbourne VCCC,<br>Bryant, Penelope; The Royal Children's Hospital Melbourne, Department of General Medicine; University of Melbourne VCCC, Department of Paediatrics<br>Babl, Franz; The Royal Children's Hospital Melbourne, Emergency Department; University of Melbourne VCCC, Department of Paediatrics<br>Ibrahim, Laila; The Royal Children's Hospital Melbourne; Murdoch Children's Research Institute<br>Hopper, Sandy; The Royal Children's Hospital Melbourne, Emergency Department<br>McNab, Sarah; The Royal Children's Hospital Melbourne, General Medicine; University of Melbourne VCCC, Department of Paediatrics<br>Donath, Susan; Murdoch Children's Research Institute, Clinical Epidemiology and Biostatistics Unit<br>Davidson, Andrew; The Royal Children's Hospital Melbourne; University of Melbourne VCCC
**Primary Subject Heading**:	Paediatrics
Secondary Subject Heading:	Emergency medicine, Infectious diseases
Keywords:	Pyelonephritis < NEPHROLOGY, Urinary tract infections < UROLOGY, Paediatric A&E and ambulatory care < PAEDIATRICS, Paediatric infectious disease & immunisation < PAEDIATRICS

SCHOLARONE™
Manuscripts

I, the Submitting Author has the right to grant and does grant on behalf of all authors of the Work (as defined in the below author licence), an exclusive licence and/or a non-exclusive licence for contributions from authors who are: i) UK Crown employees; ii) where BMJ has agreed a CC-BY licence shall apply, and/or iii) in accordance with the terms applicable for US Federal Government officers or employees acting as part of their official duties; on a worldwide, perpetual, irrevocable, royalty-free basis to BMJ Publishing Group Ltd ("BMJ") its licensees and where the relevant Journal is co-owned by BMJ to the co-owners of the Journal, to publish the Work in this journal and any other BMJ products and to exploit all rights, as set out in our licence.

The Submitting Author accepts and understands that any supply made under these terms is made by BMJ to the Submitting Author unless you are acting as an employee on behalf of your employer or a postgraduate student of an affiliated institution which is paying any applicable article publishing charge ("APC") for Open Access articles. Where the Submitting Author wishes to make the Work available on an Open Access basis (and intends to pay the relevant APC), the terms of reuse of such Open Access shall be governed by a Creative Commons licence – details of these licences and which Creative Commons licence will apply to this Work are set out in our licence referred to above.

Other than as permitted in any relevant BMJ Author's Self Archiving Policies, I confirm this Work has not been accepted for publication elsewhere, is not being considered for publication elsewhere and does not duplicate material already published. I confirm all authors consent to publication of this Work and authorise the granting of this licence.

For peer review only

Defining complicated urinary tract infection and route of antibiotics in children attending Emergency: the Melbourne RUPERT clinical score – a cohort study

Barry T Scanlan MB BCh BAO[a,f], Laila F Ibrahim MB BCh BAO[a,f], Franz E Babl MD[a,b,f], Sandy M Hopper FRACP[a,b,f], Sarah McNab PhD[a,c,f], Susan Donath[f], Andrew Davidson MD [a,e,f], Penelope A Bryant PhD[a,c,d,f,g]

Affiliations
a Department of Paediatrics, University of Melbourne
b Emergency Department, The Royal Children's Hospital
c Department of General Medicine, The Royal Children's Hospital
d Infectious Diseases Unit, Department of General Medicine, The Royal Children's Hospital
e Department of Anaesthesia, The Royal Children's Hospital
f Murdoch Children's Research Institute
g Hospital-In-The-Home Department, The Royal Children's Hospital
50 Flemington Road, Parkville, Victoria 3052, Australia

Corresponding author
A/Prof. Penelope A Bryant
Department of General Medicine, The Royal Children's Hospital Melbourne
50 Flemington Road, Parkville, VIC 3052, Australia
Email: penelope.bryant@rch.org.au
Tel: +613 93455522 Fax: +613 9345 6667

Key words
urinary tract infection, pyelonephritis, antibiotics, paediatric A&E and ambulatory care, paediatric infectious disease and immunisation

Word count: 2979

For peer review only

For peer review only - http://bmjopen.bmj.com/site/about/guidelines.xhtml

Abstract

Objectives: Most children with uncomplicated urinary tract infections (UTI) can be managed with oral antibiotics. However, identifying those likely to fail oral and need intravenous (IV) antibiotics due to complicating features at presentation is challenging. We aimed to derive, validate and test a score to guide initial antibiotic route.

Design: This cohort study enrolled children both prospectively and retrospectively. Patients were divided into 2 groups based on IV or oral antibiotic route after 24 hours, including if they switched. Children with confirmed UTI were used to derive then validate the score, comparing complicating clinical features between the 2 groups. Combinations of significantly differentiating features generated receiver operating characteristic curves and the optimal cut-off for IV antibiotic use was selected.

Setting: The Emergency Department (ED) of a tertiary paediatric hospital.

Participants: All children aged 3 months–17 years with likely UTI were eligible, and included if they fulfilled diagnostic criteria for UTI.

Outcome measures: The effectiveness of the derived clinical score to differentiate patients at presentation who had complicated UTI requiring ongoing IV antibiotics.

Results

There were 1,240 patients, of whom 167 children aged 12m–11y with confirmed UTI comprised the derivation cohort. The combination of features that performed optimally (area under curve 0.85, 95%CI 0.79-0.91) were: rigors, urological abnormality, pyrexia (≥38°C), emesis, recurrent (≥3) UTI, tachycardia: the RUPERT score (one point each, maximum 6). A score ≥3 accurately classified route of antibiotics after 24 hours for 80% patients (sensitivity 77%, specificity 81%). For the 168 patients in the validation cohort, the score accurately classified 76% (sensitivity 67%, specificity 78%). The score tested well in 'probable' UTI and adolescents, and less well in infants.

Conclusion: The Melbourne RUPERT score provides the first standardised, easy-to-use score to aid clinicians in deciding route of antibiotics for more complicated UTI in children. It now needs prospective validation.

For peer review only - http://bmjopen.bmj.com/site/about/guidelines.xhtml

Article Summary

Strengths and limitations of this study

- The study design builds on the existing evidence and creates a standardised approach where evidence is lacking.
- The study deliberately incorporates a broad range of children with UTI to offer straightforward, clinical guidance for which patients with complicating features may benefit from initial IV antibiotic treatment.
- The methods ensured internal validation and testing on different patient cohorts.
- The study has the potential limitation that in the absence of an accepted gold standard to determine route of treatment, we have accepted that the treating clinicians made the correct judgement after 24 hours.
- The single centre study design may not be reflective of all populations or universal practice and the score therefore needs prospective multicentre validation.

Funding statement

This study was funded by a grant from the RCH Foundation (RCHF 2016-718), which also in part supported BTS. PAB was supported in part by a Melbourne Campus Clinician-Scientist Fellowship, Melbourne, Australia and in part by a Medical Research Futures Fund Investigator Grant (MRF1197970). FEB was supported in part by a grant from the RCH Foundation and a Melbourne Campus Clinician-Scientist Fellowship, Melbourne, Australia and a National Health and Medical Research Council (NHMRC) Practitioner Fellowship, Canberra, Australia. The emergency research group, MCRI, is in part supported by an NHMRC Centre for Research Excellence Grant for Paediatric Emergency Medicine, Canberra, Australia and the Victorian government infrastructure support program.

Competing interests statement

All authors have indicated they have no financial relationships relevant to this article to disclose. The funding bodies do not have any authority in design and conduct of the study; collection, management, analysis, and interpretation of the data; and preparation, review, or approval of the manuscript.

Author Contributions

Dr Scanlan was involved in the design and co-ordinated the study, carried out the initial and subsequent data analysis, drafted the initial manuscript, revised subsequent drafts and approved the final manuscript as submitted. A/Prof Bryant, Prof Babl, Prof Davidson, Dr Ibrahim, Dr McNab and A/Prof Hopper were involved in the concept and design of the study, provided input into data analysis, reviewed and revised the manuscript and approved the final draft. A/Prof Donath was involved in the design of the study, advised on statistical analysis, revised and approved the final manuscript as submitted. All authors take responsibility for the integrity of the data and the accuracy of the data analysis. All authors approved the final manuscript.

Acknowledgments

We would like to acknowledge the participation of patients and families at RCH and clinical staff in the ED.

For peer review only - http://bmjopen.bmj.com/site/about/guidelines.xhtml

INTRODUCTION

Urinary tract infections (UTI) are common in children, with a prevalence rate in by the age 7 years of 1-2% in boys and 7-8% in girls[1 2]. Antibiotics are the mainstay of treatment, and most children are successfully treated with oral antibiotics. The decision between IV and oral is important: not using IV antibiotics when they are needed risks clinical deterioration, while overuse places unnecessary burden on children and the healthcare system. Systematic reviews have concluded that oral antibiotics are sufficient for both lower UTI[3] and uncomplicated upper UTI[4], including with fever, and these recommendations have been incorporated into American Academy of Pediatrics (AAP)[5] and UK National Institute for Health and Care Excellence (NICE) guidelines[6]. For complicated UTI, intravenous (IV) antibiotics are often – but not always – used. There is no consensus on the definition of complicated UTI: definitions variably include multiple factors related to past history and current presentation that may result in divergence from typical management. It is challenging to identify which children with a variety of complicating features at presentation should receive initial IV antibiotics because trials have variably excluded those with urological abnormality[7-10], previous UTI[7 8], vomiting[8 10], dehydration[8], or prior oral antibiotics[7-9], so recommendations are less robust.

In a previous study at our hospital, 72% children presenting to the Emergency Department (ED) had at least one of these complicating features and 28% received IV antibiotics[11]. Although there is no universal definition of complicated UTI, preliminary data showed that with increased number of complicating features, there was increased likelihood of initial IV antibiotic use[11]. When several factors inform treatment choice, clinical scores can help by combining important features[12]. We hypothesized that combining complicating features of UTI would generate a clinical threshold to determine route of antibiotics, and could provide an approach to defining complicated UTI.

We aimed to derive, validate and test a score that combines complicating clinical features and

For peer review only - http://bmjopen.bmj.com/site/about/guidelines.xhtml

incorporates existing evidence to guide choice of initial route of antibiotics in ED in children with UTI.

For peer review only

For peer review only - http://bmjopen.bmj.com/site/about/guidelines.xhtml

METHODS

Study design: A clinical population of consecutive patients presenting to ED with UTI was used to derive, validate and test a clinical score for defining complicated UTI and initial route of antibiotics.

Setting: The study was conducted in ED at The Royal Children's Hospital Melbourne, a tertiary paediatric hospital, from May 2016–March 2018.

Inclusion/exclusion criteria: All children aged 3 months–17 years inclusive with likely UTI in ED were eligible, and included consecutively if they fulfilled NICE diagnostic criteria[6] for symptoms and urinalysis (box 1). Patients were enrolled both prospectively to ensure integrity of the information collected, and also retrospectively to include sufficient patients to validate the findings. Patients were excluded if they were treated in ED via superseding pathways: severe infection (e.g. sepsis or meningitis) or immunodeficiency (e.g. febrile neutropenia or post renal transplant).

Clinical procedure and patient outcomes: After consent, data were collected using a standardised case record form during ED assessment for patients enrolled prospectively, and included demographics, clinical features prior to and in ED (table 1), previous history including urological abnormality (defined in box 1) and urine results. Clinical observations recorded were fever ≥38°C, tachycardia (heart rate >95th centile by age[13]), tachycardia when afebrile, and hypotension (systolic blood pressure <5th centile by 50th height centile[14]). Prospective data collected were compared with the electronic medical record and on determining a high degree of concordance, the same data were collected from retrospective participants using the same criteria. The concordance enabled missing clinical features in the retrospective clinical record review to be presumed absent. The decision to start IV or oral antibiotics was made by an

For peer review only - http://bmjopen.bmj.com/site/about/guidelines.xhtml

experienced ED physician (at least registrar or fellow level). Institutional empiric guidelines for IV antibiotics for UTI recommend gentamicin 24 hourly and benzylpenicillin 6 hourly. Ongoing antibiotic route was re-assessed at 24 hours for patients in hospital. Patients discharged from ED on oral antibiotics were presumed to remain on oral unless they re-presented to our hospital and were switched to IV. To test the assumption they were not re-presenting to other hospitals, we contacted all prospective patients within 7 days, and none re-presented to other hospitals. Data were entered into REDCap (Research Electronic Data Capture[15] at Murdoch Children's Research Institute).

Standard for assessing treatment: There is no gold standard to determine which children with UTI need IV antibiotics. A standard for assessing appropriate antibiotic route was defined using existing evidence and published methodology. For a UTI clinical score to be useful, lower and upper UTI should be included as the distinction can be challenging in young children. Existing studies show children with lower UTI and upper uncomplicated UTI do not need IV antibiotics[3 4]. For patients excluded from those studies – those with complications of vomiting, dehydration, urological abnormality, previous UTI, or oral antibiotics prior to presentation – a published method for establishing a standard for appropriate route was applied: 'the route of ongoing antibiotic treatment after review by 24 hours is likely to be appropriate based on ongoing symptom progression, even if antibiotics were administered via the other route at presentation'[16]. Therefore, if a patient on IV antibiotics was reviewed within 24 hours, and switched to oral antibiotics, they were determined to have only needed oral antibiotics initially (box 1). Likewise, if a patient treated with oral antibiotics re-presented within 24 hours and was started on IV, they were determined to have needed IV antibiotics initially. Based on this standard, patients were allocated to one of two groups: 'Oral antibiotics applicable' (no complicating features[4] *or* complicating features and on oral after 24 hours) or 'Likely to fail oral antibiotics' (complicating features and on IV after 24 hours).

For peer review only - http://bmjopen.bmj.com/site/about/guidelines.xhtml

Study cohorts: The derivation, validation and test cohorts were:

Derivation cohort: To determine which complicating features are important for a clinical prediction score, patients were included aged 12 m–11y with confirmed UTI – NICE diagnostic criteria plus positive urine or blood culture[17] (box 1). This age range was defined because patients with UTI under the age of 12 months are frequently managed differently[18], our previous study showed large variation under 12 months[19], and studies of childhood UTI characteristically have an upper limit of 12 years[20].

Validation cohort: To assess whether the score works in a separate group defined in the same way, the 12m-11y age group with confirmed UTI was divided in half into the derivation and validation cohorts by assigning patients alternately by date of presentation. Alternate assignation avoided an external change during the study having a biased effect on one group.

Test cohorts: The score was tested on additional patient groups: 1) age 12m-11y with probable (not confirmed) UTI[17], 2) age 3m-11m, and 3) age 12y-17y.

Statistical analysis: Univariate analysis was used to compare clinical features that were different between the 'Oral antibiotics applicable' and 'Likely to fail oral antibiotics' groups. For categorical data Fisher Exact test was used and for continuous data Student t test. Odds ratios (OR) and 95% confidence intervals (95%CI) were calculated and $p<0.05$ was considered significant. Each differentiating feature between the two groups was converted to a binary score of 0 (absent) or 1 (present). Different combinations of these features were added and used to generate receiver operating characteristic (ROC) curves. Scores for each feature were weighted (increased to 2) to determine whether that improved performance. The score performance with different cut-offs for identifying those likely to fail oral antibiotics was assessed as follows: 1) high ROC area under the curve (AUC); 2) most accurate assignment of patients to the groups 'Oral antibiotics applicable' and 'Likely to fail oral antibiotics'; and 3) sensitivity and

For peer review only - http://bmjopen.bmj.com/site/about/guidelines.xhtml

For peer review only

specificity. The combination of features that had the highest overall results in all of these domains was used to derive a clinical score. Statistical analysis used Stata/IC version 15.1.

Patient and public involvement: *There was no patient or public participation in the study design.*

Ethics: This study had ethics approval from RCH Human Research Ethics Committee (HREC36255). There was no patient or public participation in the study design.

For peer review only

RESULTS

Over 22 months, 1,438 patients had a diagnosis of UTI, of which 46 were excluded due to miscoding or other reasons (figure 1a). 1,392 children were treated as having a UTI in ED. Of these, 152 were excluded for not fulfilling NICE criteria (box 1) or other exclusions, resulting in 1,240 patients with UTI. Of these 1,240, 276 (22%) were aged 3m-11m, 831 (67%) were 12m-11y and 133 (11%) were 12y-17y.

Clinical features and treatment of derivation cohort

Of 831 patients aged 12m-11y, 335 had confirmed UTI (box 1). Half (167) were assigned to the derivation cohort, and 168 to the validation cohort, with no difference between groups in triage category and hospital admission (data not shown). The treatment allocations at 24 hours were: derivation cohort 35 (21%) IV and 132 (79%) oral; validation cohort 47 (28%) IV and 121 (72%) oral. In the derivation cohort, there were 43 children with no complicating features and 89 with complicating features who were on oral by 24 hours (80 continuing and 9 de-escalated from IV) totalling 132 in the 'Oral antibiotics applicable' group. Of the 9 who de-escalated, 8 had only a 24-hourly dosed antibiotic, and 1 additionally had multi-dose antibiotics per day. There were 35 children with complicating features and on IV after 24 hours (32 continuing and 3 escalated from oral) and these were assigned to the 'Likely to fail oral antibiotics' group (figure 1b).

Derivation of clinical score

Multiple patient characteristics and clinical features that have been variably included in definitions of complicated UTI or excluded from definitions of uncomplicated UTI were compared between the 'Oral antibiotics applicable' and 'Likely to fail oral antibiotics' groups. Ten features significantly differentiated between the groups (either recorded in ED or reported in the previous 24 hours): fever ≥38°C, rigors, vomiting, lethargy, flank pain/tenderness, dysuria, urological abnormality (known anatomical or functional anomaly of any part of the urological

For peer review only

tract), previous UTI, documented recurrent (≥3) UTI, any tachycardia, tachycardia when afebrile (table 1). For features that were variants of the same measure, the one that differentiated the groups more significantly was selected: 'any tachycardia' differentiated more than 'tachycardia when afebrile', and 'recurrent (≥3) UTI' differentiated more than 'any previous UTI'. The ROC curve of this eight-feature score had a high AUC of 0.86 (95%CI 0.81-0.92). Features were serially removed from this score and different cut-offs were tested to compare performance in terms of AUC, correct identification of 'Likely to fail oral antibiotics', sensitivity and specificity. Score performance did not deteriorate until the number of features was below six (supplemental table 1 and figure 2a). There was expected overlap between some features: for example, in the total population, 217 (18%) children had both fever and tachycardia, while 92 (7%) had tachycardia when afebrile and 444 (36%) had fever without tachycardia. Likewise, 130 (6%) had both urological abnormality and recurrent UTI, while 214 (10%) patients had urological abnormality without recurrent UTI and 261 (12%) patients had recurrent UTI without documented abnormality.

The Melbourne RUPERT score

Using six of the eight discriminating features, the combination that performed best was rigors, urological abnormality, pyrexia, emesis, recurrent (>2) UTI and tachycardia, which can be remembered using the acronym RUPERT. Applying different weights did not affect score performance (data not shown). The score had a ROC AUC of 0.85 (95%CI 0.79-0.91) (figure 2a). Next, a RUPERT score cut-off was determined, above which patients should be considered for IV antibiotics due to having more complicated UTI, and below which oral antibiotics could be used. A cut-off of 3 – patients with a score of 3 or more considered for IV antibiotics – correctly classified the route of antibiotics after 24 hours for 80% of patients, with sensitivity 77%, specificity 81% (supplemental table 1, figure 3). Applying a cut-off of 3, IV antibiotics would have been recommended for 52 (31%) patients and oral for 115 (69%) patients. This

For peer review only - http://bmjopen.bmj.com/site/about/guidelines.xhtml

For peer review only

threshold for use of IV may act as a surrogate for identifying more complicated UTI. A cut-off of 4 correctly classified 81% of patients, with sensitivity 31%, specificity 95%. With a cut-off of 4, IV antibiotics would have been recommended for 18 (11%) patients and oral for 149 (89%). Increasing the cut-off from 3 to 4 would result in increased patients failing oral treatment from 8 (5%) to 24 (14%). For the 9 who de-escalated from IV to oral by 24 hours, 2 had a score of 1 (both recurrent UTI), 4 scored 2 (2 fever and vomiting, 1 fever and tachycardia, 1 fever and urological abnormality), 1 scored 3 (fever, rigors and vomiting), and 2 scored 4 (both fever, vomiting, recurrent UTI and urological abnormality – 1 with neurogenic bladder, 1 with pyeloplasty).

To ensure the derived score performed rationally, we examined its use on afebrile patients since only a minority would be expected to need IV antibiotics (and these patients would need 3 other features to reach the threshold). Only 3 patients were still on IV antibiotics at 24 hours: 2 with a RUPERT score of 3 (both with severe urological abnormalities – neurogenic bladder, mitrofanoff, and severe VUR – and recurrent UTI, 1 also had vomiting and the other also had rigors). The other patient had a RUPERT score of 1 (recurrent UTI), and had had 7 days of failed oral treatment, so was treated with IV while waiting for culture results at 48 hours, at which point was switch to a different oral choice for a resistant pathogen. It was therefore deemed to work appropriately.

For peer review only

Validation of the RUPERT score

The score was validated on 168 patients with the same criteria (age 12m-11y with confirmed UTI)(supplemental table 2). Applying the same standard, by 24 hours 121 (72%) were receiving oral antibiotics and 47 (28%) IV antibiotics. The RUPERT score had a similarly high AUC of 0.80 (95%CI 0.73-0.88) (figure 2b) in this cohort. A score cut-off of 3 correctly classified 76% of patients (sensitivity 67%, specificity 78%). Applying a cut-off of 3, IV antibiotics would have been suggested for 51 (30%) patients oral antibiotics for 117 (70%) patients. With a cut-off of 3

For peer review only - http://bmjopen.bmj.com/site/about/guidelines.xhtml

in the validation cohort, 10 (6%) patients recommended for oral antibiotics would fail (table 2).

Testing the RUPERT score on other cohorts

1) *Age 12m-11y with probable UTI:* There were 498 patients in this group with a diagnosis of UTI that was likely, but not culture-confirmed[17]. Applying the score resulted in an AUC of 0.82 (95%CI 0.76-0.87), and the cut-off of 3 correctly assigned 82% of patients (sensitivity 54%, specificity 85%)(table 2)(figure 2c and supplemental table 3).
2) *Age 12y-17y with confirmed or probable UTI:* There were 133 patients in this adolescent group. Applying the score resulted in an AUC of 0.86 (95%CI 0.79-0.92), and the cut-off of 3 correctly assigned 86% of patients (sensitivity 57%, specificity 86%).
3) *Age 3m-11m with confirmed or probable UTI:* There were 276 patients in this infant group. Applying the score resulted in an AUC of only 0.58 (95%CI 0.51-0.65), and the cut-off of 3 correctly assigned 64% patients - the lowest proportion in any group (sensitivity 43%, specificity 72%).

There was no difference between the performance of the score in the 12m-17y age range between the 276 patients where data were collected prospectively (AUC 0.84, 95%CI 0.79-0.87), and the 688 patients where collection was retrospective (AUC 0.83, 95%CI 0.79-0.87).

For peer review only

DISCUSSION

The Melbourne RUPERT score is applicable to a broad range of children presenting to hospital with UTI. By including all children presenting to ED with UTI, it avoids the need to determine whether a child fits an existing definition of complicated UTI. It offers straightforward, clinically-relevant guidance for assessing which patients with complicating features of UTI may benefit from initial IV antibiotic treatment that is currently not articulated in major guidelines. It also offers a potential new approach to defining complicated UTI: rather than a one-size-fits-all

For peer review only - http://bmjopen.bmj.com/site/about/guidelines.xhtml

definition of specific features, it can be considered as a threshold of multiple complicating features which result in a different management pathway. The definition of complicated UTI is widely variable between studies and guidelines with a range of different host conditions and types of severity falling under the same umbrella term. The RUPERT score solves the problem of separately categorising these often overlapping presentations, by using patients' own various complicating features to build to a threshold for recommending IV antibiotics, regardless of what those individual features are. Each of the features in the score is easily assessable, well-defined and objective and the score does not require patients to verbalise symptoms. Additionally, it does not rely on invasive blood tests or waiting for urine culture results. The score performed well in pre- and primary school age children and adolescents with confirmed and probable UTI. It less accurately identified infants under 12 months receiving oral antibiotics. Although, this could be because the score does not perform well in infants, it is more likely due to the frequent initiation and/or treatment beyond 24 hours with IV antibiotics in this age group, without a clear clinical indication. There is no pathophysiological reason that infants aged 3 to 11 months should be different to older children, but both our data (45% IV initiation for all UTI in 3-11m, versus 28% in the derivation/validation cohorts) and previous studies have shown higher IV rates in this age group without clear clinical reasons[18 19]. This therefore would benefit from prospective application and testing, and could help reduce unnecessary IV in this age group.

Using a RUPERT score cut-off of 3 provides a higher sensitivity to reduce oral antibiotic failures, at the expense of considering more patients for IV antibiotics. Alternatively, a cut-off of 4 substantially increases the number of patients recommended for oral antibiotics but reduces the sensitivity. Like most clinical scores, it performs well at the extremes and the mid-range involves a balance between sensitivity and specificity[12]. Scores in the mid-range give clinicians information about the need for monitoring in the first 24 hours of treatment. Scores in the lower range reassure that even with features potentially associated with sepsis (rigors, tachycardia), in

For peer review only - http://bmjopen.bmj.com/site/about/guidelines.xhtml

isolation do not necessitate IV antibiotic use. We did not seek to exclude features that were more likely to occur together (e.g. fever and tachycardia, urological abnormality and recurrent UTI), as co-occurrence may signal increased severity of illness or risk of rapid damage to the kidney, when the IV antibiotics would be appropriate.

The lack of a gold standard required the development of one from a combination of existing evidence (which is robust due to multiple RCTs contributing), and where evidence was limited, using published methodology derived from early clinical review. Although the methodology was devised for cellulitis, we have previously shown that patients with UTI who received less than 24 hours' IV antibiotics were more similar to patients who received only oral antibiotics than patients who received 2-3 days' IV antibiotics[Determining the Management of Children with Acute UTI/Pyelonephritis Who Do Not Fit Current Management Recommendations. Scanlan BT, Ibrahim LI, Hopper SM, McNab S, Babl FE, Bryant PA. IDWeek, San Francisco, USA 2018; Open Forum Inf Dis Nov 2018; 5 (Supp 1) https://doi.org/10.1093/ofid/ofy210.1349]. This adds weight to the supposition for the devised gold standard that this group only needed oral antibiotics, although without a cast-iron clinical marker for 'need for IV', this could not be guaranteed in every case. It was therefore important to ensure that clinical practice at our hospital is similar to practice elsewhere. A recent study of UTI at 36 US hospitals between 2010 and 2016 reported that in children aged 3 to 24 months, 35% were treated with IV antibiotics[21]. This is similar to 39% use in this age group at our hospital (data not shown separately)[11].

Guidelines have proved useful in standardising management of UTI, and major international guidelines developed by NICE[6] and AAP[5] are clear in many areas of diagnosis and management (e.g. need for imaging). However, both guidelines leave treatment with IV antibiotics more flexible, suggesting that it should be based on 'practical considerations' (AAP) and 'if oral antibiotics cannot be used' (NICE). The range of conditions which broadly and inconsistently

For peer review only - http://bmjopen.bmj.com/site/about/guidelines.xhtml

fall under the wide definition of complicated UTI means that it has consistently been excluded from trials, guidelines and even retrospective studies of management. Clinicians are left trying to determine if their patient really has a complicated UTI, what exactly that means and how to manage the child in front of them. By defining patients not previously fitting within evidence-based recommendations, the RUPERT score adds clinical context to practical considerations and standardises recommendations for when oral antibiotics are more likely to fail. It allows for an individualised approach by using an individual child's own clinical features rather than trying to impose a particular definition of complicated UTI, which is additionally lacking evidence for management .

Incorporating a clinical score into management decisions offers an aid to reducing variation in care. Paediatric scores that have been successfully developed and implemented include the Westley score[22] for croup, the Pediatric Asthma Severity Score (PASS)[23] and the Melbourne ASSET score for cellulitis.[16] In addition to reducing variation in care, a clinical score can standardise route of antibiotics in trials, important for generalising outcomes. For example, in one study describing the outcomes of outpatient parenteral antibiotic treatment for UTI, the authors admitted that the patients could have been treated with oral antibiotics[18]. This clinical score could be used for inclusion/exclusion criteria in studies (e.g. whether participants exceed a score cut-off) and/or to describe the relative clinical severity of study subjects by recording their RUPERT score[24].

The strengths of this score, in addition to being the first to attempt to provide standardised guidance where evidence is lacking, are that it is easy to use and does not require invasive tests or waiting for culture results. In addition, by being applicable to all children with UTI because it incorporates existing strong evidence for those with uncomplicated UTI, clinicians do not need to determine whether a patient has complicated UTI before applying it. There are several

For peer review only - http://bmjopen.bmj.com/site/about/guidelines.xhtml

For peer review only

limitations, the main one being that it has not yet been tested prospectively in other settings and populations. Although the heterogeneity of our population provides some assurance about generalizability, the score could perform differently in other populations that are more homogenous or heterogenous in a different way. Prospective validation in other settings is therefore essential. Another potential limitation is that we have accepted that the treating clinicians made the correct judgement at 24 hours on treatment route. While these decisions are made by senior doctors, practice varies between clinicians. Review of medical records, however, did not suggest any unusual practice, or that many patients receiving oral antibiotics returned. Using this score does not negate the possibility of overtreating some patients with IV and undertreating some with oral. However, it aims to be a starting point for standardising the rationale for IV antibiotics, while allowing institutional flexibility to determine the treatment outcomes for which they are aiming.

CONCLUSION

The Melbourne RUPERT score is an easy-to-use and reproducible clinical score for UTI management in children. It is inclusive of all children with UTI, by incorporating robust evidence for children with uncomplicated UTI, and a new approach to defining patients with complicated UTI to standardise risk stratification and treatment recommendations, both for clinical practice and research. As with any decision-support tool, the aim is to offer a guide rather than completely replace clinical judgement for individual patients. The score will now benefit from external validation, refinement and impact analysis.

For peer review only - http://bmjopen.bmj.com/site/about/guidelines.xhtml

References

1. Shaikh N, Morone NE, Bost JE, et al. Prevalence of urinary tract infection in childhood: A meta-analysis. *Pediatric Infectious Disease Journal* 2008;27(4):302-08. doi: 10.1097/INF.0b013e31815e4122
2. Sood A, Penna FJ, Eleswarapu S, et al. Incidence, admission rates, and economic burden of pediatric emergency department visits for urinary tract infection: data from the nationwide emergency department sample, 2006 to 2011. *Journal of pediatric urology* 2015;11(5):246. e1-46. e8.
3. Fitzgerald A, Mori R, Lakhanpaul M, et al. Antibiotics for treating lower urinary tract infection in children. *Cochrane Database Syst Rev* 2012(8):CD006857. doi: 10.1002/14651858.CD006857.pub2 [published Online First: 2012/08/17]
4. Strohmeier Y, Hodson EM, Willis NS, et al. Antibiotics for acute pyelonephritis in children. *Cochrane Database of Systematic Reviews* 2014(7):CD003772. doi: https://dx.doi.org/10.1002/14651858.CD003772.pub4
5. Roberts KB, Downs SM, Finnell SME, et al. Urinary tract infection: Clinical practice guideline for the diagnosis and management of the initial UTI in febrile infants and children 2 to 24 months. *Pediatrics* 2011;128(3):595-610. doi: 10.1542/peds.2011-1330
6. Baumer JH, Jones RW. Urinary tract infection in children, National Institute for Health and Clinical Excellence. *Arch Dis Child Educ Pract Ed* 2007;92(6):189-92. doi: 10.1136/adc.2007.130799
7. Hoberman A, Wald ER, Hickey RW, et al. Oral versus initial intravenous therapy for urinary tract infections in young febrile children. *Pediatrics* 1999;104(1 Pt 1):79-86.
8. Montini G, Toffolo A, Zucchetta P, et al. Antibiotic treatment for pyelonephritis in children: Multicentre randomised controlled non-inferiority trial. *British Medical Journal* 2007;335(7616):386-88. doi: 10.1136/bmj.39244.692442.55
9. Neuhaus TJ, Berger C, Buechner K, et al. Randomised trial of oral versus sequential intravenous/oral cephalosporins in children with pyelonephritis. *European Journal of Pediatrics* 2008;167(9):1037-47. doi: https://dx.doi.org/10.1007/s00431-007-0638-1
10. Bocquet N, Alaoui AS, Jais JP, et al. Randomized trial of oral versus sequential IV/Oral antibiotic for acute pyelonephritis in children. *Pediatrics* 2012;129(2):e269-e75. doi: http://dx.doi.org/10.1542/peds.2011-0814
11. Scanlan B, Ibrahim L, Hopper S, et al. 1520. Determining the Management of Children with Acute UTI/Pyelonephritis Who Do Not Fit Current Management Recommendations. *Open Forum Infectious Diseases* 2018;5(suppl_1):S471-S71. doi: 10.1093/ofid/ofy210.1349
12. Laupacis A, Sekar N. Clinical prediction rules: a review and suggested modifications of methodological standards. *Jama* 1997;277(6):488-94.
13. Bonafide CP, Brady PW, Keren R, et al. Development of heart and respiratory rate percentile curves for hospitalized children. *Pediatrics* 2013;131(4):e1150-7. doi: 10.1542/peds.2012-2443 [published Online First: 2013/03/13]
14. Haque IU, Zaritsky AL. Analysis of the evidence for the lower limit of systolic and mean arterial pressure in children. *Pediatr Crit Care Med* 2007;8(2):138-44. doi: 10.1097/01.PCC.0000257039.32593.DC [published Online First: 2007/02/03]
15. Harris PA, Taylor R, Thielke R, et al. Research electronic data capture (REDCap)--a metadata-driven methodology and workflow process for providing translational research informatics support. *J Biomed Inform* 2009;42(2):377-81. doi: 10.1016/j.jbi.2008.08.010 [published Online First: 2008/10/22]

For peer review only

16. Ibrahim LF, Hopper SM, Donath S, et al. Development and Validation of a Cellulitis Risk Score: The Melbourne ASSET Score. *Pediatrics* 2019 doi: 10.1542/peds.2018-1420 [published Online First: 2019/01/05]
17. Basmaci R, Vazouras K, Bielicki J, et al. Urinary Tract Infection Antibiotic Trial Study Design: A Systematic Review. *Pediatrics* 2017;140(6) doi: 10.1542/peds.2017-2209 [published Online First: 2017/12/01]
18. Gauthier M, Chevalier I, Sterescu A, et al. Treatment of urinary tract infections among febrile young children with daily intravenous antibiotic therapy at a day treatment center. *Pediatrics* 2004;114(4):e469-76.
19. Scanlan B, Ibrahim L, Quinlan C, et al. Why are children with febrile urinary tract infection/pyelonephritis treated with intravenous antibiotics? International Paediatric Nephrology Society Association Meeting. Germany, 2017.
20. Baker PC, Nelson DS, Schunk JE. The addition of ceftriaxone to oral therapy does not improve outcome in febrile children with urinary tract infections. *Archives of Pediatrics & Adolescent Medicine* 2001;155(2):135-9.
21. Chaudhari PP, Monuteaux MC, Bachur RG. Emergency Department Revisits After an Initial Parenteral Antibiotic Dose for UTI. *Pediatrics* 2018;142(3) doi: 10.1542/peds.2018-0900 [published Online First: 2018/08/23]
22. Westley CR, Cotton EK, Brooks JG. Nebulized racemic epinephrine by IPPB for the treatment of croup: a double-blind study. *Am J Dis Child* 1978;132(5):484-7. [published Online First: 1978/05/01]
23. Gorelick MH, Stevens MW, Schultz TR, et al. Performance of a novel clinical score, the Pediatric Asthma Severity Score (PASS), in the evaluation of acute asthma. *Acad Emerg Med* 2004;11(1):10-8. [published Online First: 2004/01/08]
24. clinical.trials.gov. Single Dose Intravenous Antibiotics for Complicated Urinary Tract Infections in Children (CHOICE UTI): 2021; 2021 [Available from: https://clinicaltrials.gov/ct2/show/NCT04876131?recrs=a&cond=uti&age=0&draw=3&rank=11] accessed 15/05/2023 2023.
25. Urinary tract infectionin under 16s: diagnosis and management, clinical guideline CG54.: United Kingdom National Institute for Health and Care Excellence; 2017 [Available from: https://www.nice.org.uk/Guidance/CG54 accessed 21.06.2018 2018.

For peer review only - http://bmjopen.bmj.com/site/about/guidelines.xhtml

Box 1: Definitions of groups

1) Definitions for eligibility
NICE clinical criteria* for diagnosis of UTI at presentation to ED

Symptoms: Age <2 years: fever, rigor, vomiting, flank tenderness, poor feeding, irritability, offensive urine, gross haematuria or failure to thrive
Age ≥2 years: fever, rigor, vomiting, abdominal pain, flank pain/tenderness, dysfunctional voiding, change in urinary continence, dysuria, gross haematuria, frequency, offensive urine or cloudy urine

AND (collected by clean catch, mid-stream urine, in/out catheter or supra-pubic aspirate)

Urinalysis: Abnormal urinary dipstick leucocyte esterase >1+ or nitrite positive
OR
White cell count (WCC) >10 x10^6/l in uncentrifuged urine
OR
Bacteriuria

opinion

2) Definitions for derivation/validation/test cohorts
Confirmed UTI†

Patients who met NICE clinical criteria for diagnosis and who were treated as having UTI
AND

Culture: Positive urine culture with no more than 2 species of microorganisms >10^7 CFU/L
OR
Positive blood culture and no other recognised cause

Probable UTI

Patients who met NICE clinical criteria for diagnosis and who were treated as having UTI but NOT fulfilling research criteria recommendations for culture-based 'confirmed' UTI.

3) Definitions for group classification

'Likely to fail oral antibiotics' – complicating features (vomiting, dehydration, urological abnormality*, previous UTI, or oral antibiotics prior to presentation) *and* on IV after 24 hours

'Oral antibiotics applicable' – no complicating features, or complicating features and on oral after 24 hours

*Urological abnormalities included any known anatomical or functional abnormality of the urological tract, as deemed relevant by the physician in the management of the current UTI. These ranged from moderate/severe (e.g. ureteric obstruction, duplex kidneys with vesico-ureteric reflux (VUR), grades III-V VUR, grades III-IV hydronephrosis, neurogenic bladder, post pyeloplasty and post ureteric implantation), to mild (e.g. post hypospadias repair, grade I-II VUR, grade I-II hydronephrosis, phimosis with balanitis).

UTI – urinary tract infection, ED – emergency department, CFU – colony forming units, *NICE guidelines[25] †Trial design recommendations[25]

For peer review only - http://bmjopen.bmj.com/site/about/guidelines.xhtml

Figure legend

Figure 1. Patient assignment to a) derivation, validation and test cohorts and b) within the derivation cohort to ‘Oral antibiotics applicable’ and ‘Likely to fail oral antibiotics’ groups in the derivation cohort; UTI – urinary tract infections, ED – emergency department, IV – intravenous, y – year(s), m – month(s), h – hour(s)

Figure 2. Comparison of receiver operating characteristic curves with a) different numbers of features for the derivation cohort; b) for derivation and validation cohorts; and c) for further test cohorts; (AUC (95%CI))

Figure 3. Sensitivity and specificity at each cut-off threshold of the Melbourne RUPERT score for the derivation cohort

For peer review only

For peer review only - http://bmjopen.bmj.com/site/about/guidelines.xhtml

Table 1: Comparison between the 'Oral antibiotics applicable' and 'Likely to fail oral antibiotics' groups in the derivation cohort

	Oral antibiotics applicable No. (%) n=132	Likely to fail oral antibiotics No. (%) n=35	OR (95%CI)	p value
Age, y (mean±SD)	5.1±3.1	4.9±3.2		0.98
Female	103 (78)	26 (74)	0.8 (0.3-1.9)	0.64
Clinical features (either recorded in ED or reported in the previous 24 hours)				
Fever ≥38°C	81 (61)	32 (91)	6.7 (2.1-22)	<0.001
Rigors	8 (6)	6 (17)	3.2 (1.1-10)	0.04
Vomiting	41 (31)	18 (51)	2.4 (1.1-5)	0.03
Lethargy	19 (14)	13 (37)	3.5 (1.5-8)	0.002
Abdominal pain	61 (46)	17 (49)	1.1 (0.5-2.3)	0.8
Flank pain/tenderness	16 (12)	9 (26)	2.5 (1-6.2)	0.045
Dysfunctional voiding	5 (4)	1 (3)	0.8 (0-5.1)	0.79
Dysuria	62 (47)	8 (23)	0.3 (0.1-0.8)	0.01
Offensive urine	17 (13)	3 (9)	0.6 (0.2-2.1)	0.49
Gross haematuria	14 (11)	1 (3)	0.2 (0-1.5)	0.15
Frequency	23 (17)	2 (6)	0.3 (0-1.2)	0.08
Suprapubic tenderness	22 (17)	3 (9)	0.5 (0.1-1.6)	0.23
Previous history				
Urological abnormality*	20 (15)	17 (49)	5.3 (2.4-11.9)	<0.001
Any previous documented UTI	52 (39)	24 (69)	3.4 (1.5-7.3)	0.002
Recurrent (≥3) documented UTI	26 (20)	21 (60)	6.1 (2.8-13.5)	<0.001
Antibiotic prophylaxis (current)	6 (5)	4 (11)	2.7 (0.8-9.6)	0.13
Known resistant organisms†	19 (14)	10 (29)	2.4 (1-5.7)	0.05
Prior oral antibiotics this UTI	26 (20)	8 (23)	1.2 (0.5-2.9)	0.68
Clinical observations				
Tachycardia (any)	27 (20)	18 (51)	4.1 (1.9-9)	<0.001
Tachycardia when afebrile	4 (3)	4 (3)	4.1 (1.1-16)	0.04
Hypotension	0 (0)	0 (0)	NA	NA

OR: odds ratio, SD: standard deviation, ED: emergency department, UTI: urinary tract infection

*Urological abnormality: known anatomical or functional abnormality of the urological tract; this included any abnormality that the treating physician felt was relevant to the UTI management

†Resistant to the recommended antibiotics in the institutional guideline

For peer review only - http://bmjopen.bmj.com/site/about/guidelines.xhtml

Table 2: Effect on patient management to all cohorts aged 3 months-17 years

Cohort	Derivation 12m-11y Confirmed UTI No. (%)	Validation 12m-11y Confirmed UTI No. (%)	Test 12m-11y Probable UTI No. (%)	Test 12y-17y All UTI No. (%)	Test 3m-11m All UTI No. (%)	Final 12m-17y All UTI No. (%)
Patients	167	168	498	133	276	966
Route of initial antibiotic treatment						
Oral	124 (74)	117 (70)	421 (85)	91 (68)	153 (55)	753 (78)
IV	43 (26)	51 (30)	77 (15)	42 (32)	123 (45)	213 (22)
Route of antibiotic treatment by 24 hours						
Oral	132 (79)	138 (82)	448 (90)	112 (84)	197 (71)	830 (86)
IV	35 (21)	30 (18)	50 (10)	21 (16)	79 (29)	136 (14)
Melbourne RUPERT score assignation						
Recommend oral (score <3)	115 (69)	117 (70)	406 (82)	105 (79)	188 (68)	743 (77)
Recommend IV (score ≥3)	52 (31)	51 (30)	92 (18)	28 (21)	88 (32)	223 (23)

UTI – urinary tract infection, IV – intravenous

For peer review only

For peer review only - http://bmjopen.bmj.com/site/about/guidelines.xhtml

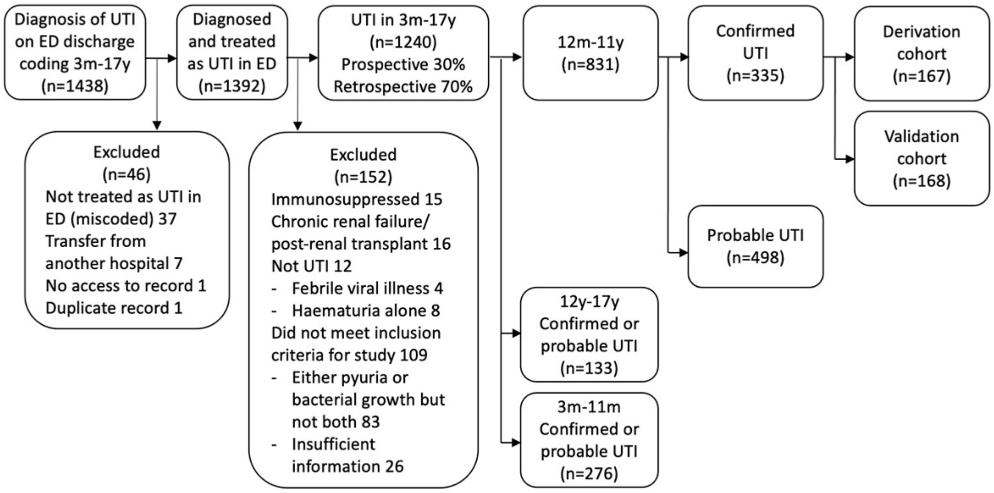

Patient assignment to a) derivation, validation and test cohorts and b) within the derivation cohort to 'Oral antibiotics applicable' and 'Likely to fail oral antibiotics' groups in the derivation cohort; UTI – urinary tract infections, ED – emergency department, IV – intravenous, y – year(s), m – month(s), h – hour(s)

149x74mm (200 x 200 DPI)

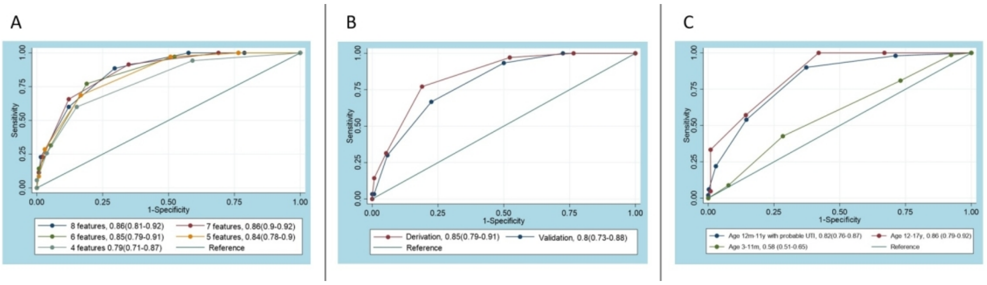

Comparison of receiver operating characteristic curves with a) different numbers of features for the derivation cohort; b) for derivation and validation cohorts; and c) for further test cohorts; (AUC (95%CI))

380x105mm (120 x 120 DPI)

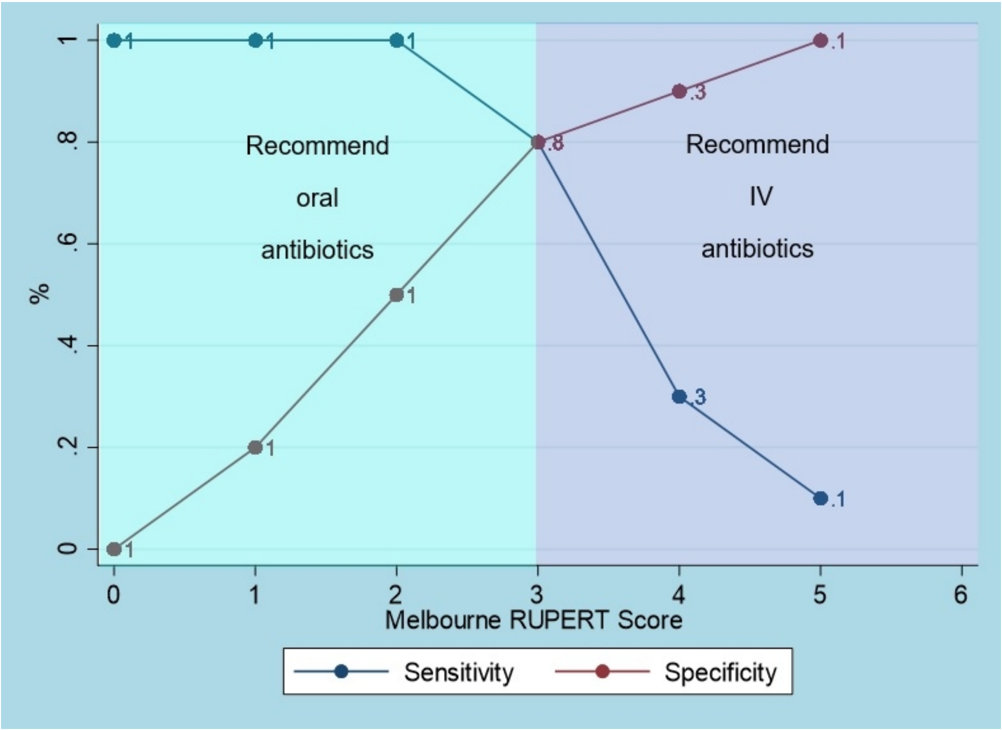

Sensitivity and specificity at each cut-off threshold of the Melbourne RUPERT score for the derivation cohort

138x100mm (144 x 144 DPI)

Supplemental table 1. Sensitivity, specificity and classification accuracy of the Melbourne RUPERT score

**Cut-off**	**Sensitivity (%)**	**Specificity (%)**	**Proportion of patients correctly classified (%)**
>=0	100	0	21
>=1	100	23	40
>=2	97	48	58
>=3	77	81	80
>=4	31	95	81
>=5	14	99	81
>5	0	100	79

For peer review only

Supplemental table 2. Comparison of clinical features in derivation and validation cohorts

	Derivation No. (%) n=167	Validation No. (%) n=168	p value
Age, y (mean±SD)	5±3.1	5.1±3.2	0.94
Female	129 (77)	138 (82)	0.27
Clinical features (prior to presentation or in the ED)			
Fever	113 (68)	106 (63)	0.38
Rigors	14 (8)	18 (11)	0.47
Vomiting	59 (35)	56 (33)	0.7
Lethargy	32 (19)	33 (20)	0.91
Abdominal pain	78 (47)	74 (44)	0.63
Flank pain/tenderness	25 (15)	31 (18)	0.39
Dysfunctional voiding	6 (4)	8 (5)	0.59
Dysuria	70 (42)	63 (38)	0.41
Offensive urine	113 (68)	106 (63)	0.13
Gross haematuria	14 (8)	18 (11)	0.99
Frequency	59 (35)	56 (33)	0.18
Suprapubic tenderness	32 (19)	33 (20)	0.51
Previous history			
Urological abnormality*	37 (22)	32 (19)	0.48
Any previous documented UTI	76 (46)	86 (51)	0.3
Recurrent (≥3) documented UTI	47 (28)	47 (28)	0.97
Antibiotic prophylaxis (current)	10 (6)	8 (5)	0.62
Known resistant organism	29 (17)	28 (17)	0.86
Prior oral antibiotics this UTI	34 (20)	28 (17)	0.43
Clinical observations			
Tachycardia (any recorded in ED)	45 (27)	39 (23)	0.43
Tachycardia when afebrile	8 (5)	5 (3)	0.39

Data presented are n (%) unless stated otherwise. SD – standard deviation, ED – emergency department, UTI – Urinary tract infection, fever – ≥38°C, y – years, SD – standard deviation

For peer review only

Supplemental table 3: Performance of the Melbourne RUPERT score in all cohorts

	ROC AUC	Score	Sensitivity	Specificity	Proportion of patients correctly identified	Proportion of patients assigned to consider IV
Derivation and validation cohorts						
Derivation (12m-11y confirmed UTI)	0.85	3/6	77%	81%	80%	31%
		2/6	97%	48%	58%	62%
		4/6	31%	95%	81%	11%
Validation (12m-11y confirmed UTI)	0.80	3/6	67%	78%	76%	30%
Test cohorts						
12m-11y probable UTI	0.82	3/6	54%	85%	82%	18%
12y-17y all UTI	0.86	3/6	57%	86%	81%	21%
3m-11m all UTI	0.58	3/6	43%	72%	64%	32%

ROC=receiver operator curve, AUC=area under the curve, IV – intravenous, m – months, y – years, UTI – urinary tract infection

For peer review only

Success criteria for Clinical Decision Rules[1] – check list completed for the Melbourne RUPERT score

For a Clinical Decision Rule to be considered successful, all of the following should apply:

General

- *Need:* Outlined in the introduction

Rule Derivation Methodology

- *Setting and Population:* This rule was derived using a representative population, children attending the ED, for which it is intended to be used.
- *Outcome:* As described is both clinically important and consistently assessed
- *Predictor Variables:* The predictive variables used were reliably recorded and are clinically relevant. These were based on patient history and observed vital signs in the ED, this produced a clinical score with features that are easily assessable, well-defined and objective and the score does not require the patient to verbalise symptoms. Additionally, this score does not rely on results of blood or urine tests. The score was shown to be equally reliable when the information was collected prospectively and retrospectively. Observers inputted variables prior to the creation of the score and were therefore unaware of which variables would be used.
- *Rule creation:* The rule was created incorporating best available evidence and following previously published methodology as described.

Rule Derivation Reporting

- *Performance:* Analysis including receiver operating characteristic (ROC) curves, sensitivity and specificity are clearly and prominently reported both in the text and in figures, 95% confidence intervals are reported.
- *Success:* The score offers an aid to clinical decision making as was the aim, it is easy-to-use and reproducible. While the score has been internally validated it would benefit from external validation and impact analysis as stated.
- *Reliability:* The rule performed reliably in those patients aged 12 months to 17 years old.

Reference:

1. Green SM, Schriger DL, Yealy DM. Methodologic standards for interpreting clinical decision rules in emergency medicine: 2014 update. *Ann Emerg Med.* 2014;64(3):286-291.

For peer review only

Section & Topic	No	Item	Reported on page #
**TITLE OR ABSTRACT**			
	**1**	Identification as a study of diagnostic accuracy using at least one measure of accuracy (such as sensitivity, specificity, predictive values, or AUC)	2 (Abstract)
**ABSTRACT**			
	**2**	Structured summary of study design, methods, results, and conclusions (for specific guidance, see STARD for Abstracts)	2
**INTRODUCTION**			
	**3**	Scientific and clinical background, including the intended use and clinical role of the index test	4
	**4**	Study objectives and hypotheses	4-5
**METHODS**			
Study design	**5**	Whether data collection was planned before the index test and reference standard were performed (prospective study) or after (retrospective study)	6
Participants	**6**	Eligibility criteria	6
	**7**	On what basis potentially eligible participants were identified (such as symptoms, results from previous tests, inclusion in registry)	6
	**8**	Where and when potentially eligible participants were identified (setting, location and dates)	6
	**9**	Whether participants formed a consecutive, random or convenience series	6
Test methods	**10a**	Index test, in sufficient detail to allow replication	6-7
	**10b**	Reference standard, in sufficient detail to allow replication	7
	**11**	Rationale for choosing the reference standard (if alternatives exist)	7
	**12a**	Definition of and rationale for test positivity cut-offs or result categories of the index test, distinguishing pre-specified from exploratory	8-9
	**12b**	Definition of and rationale for test positivity cut-offs or result categories of the reference standard, distinguishing pre-specified from exploratory	8-9
	**13a**	Whether clinical information and reference standard results were available to the performers/readers of the index test	7
	**13b**	Whether clinical information and index test results were available to the assessors of the reference standard	7
Analysis	**14**	Methods for estimating or comparing measures of diagnostic accuracy	8-9
	**15**	How indeterminate index test or reference standard results were handled	N/A
	**16**	How missing data on the index test and reference standard were handled	6
	**17**	Any analyses of variability in diagnostic accuracy, distinguishing pre-specified from exploratory	N/A
	**18**	Intended sample size and how it was determined	N/A
**RESULTS**			
Participants	**19**	Flow of participants, using a diagram	10 & 22 (figure 1)
	**20**	Baseline demographic and clinical characteristics of participants	10 & 25 (table 1)
	**21a**	Distribution of severity of disease in those with the target condition	10 & 22 (figure 1b)
	**21b**	Distribution of alternative diagnoses in those without the target condition	10 & 22 (figure 1b)
	**22**	Time interval and any clinical interventions between index test and reference standard	10
Test results	**23**	Cross tabulation of the index test results (or their distribution) by the results of the reference standard	10-11
	**24**	Estimates of diagnostic accuracy and their precision (such as 95% confidence intervals)	11-12
	**25**	Any adverse events from performing the index test or the reference standard	N/A
**DISCUSSION**			
	**26**	Study limitations, including sources of potential bias, statistical uncertainty, and generalisability	16-17
	**27**	Implications for practice, including the intended use and clinical role of the index test	13-17
**OTHER INFORMATION**			
	**28**	Registration number and name of registry	N/A
	**29**	Where the full study protocol can be accessed	N/A
	**30**	Sources of funding and other support; role of funders	3

For peer review only - http://bmjopen.bmj.com/site/about/guidelines.xhtml

CC BY SA

STARD 2015

AIM

STARD stands for “Standards for Reporting Diagnostic accuracy studies”. This list of items was developed to contribute to the completeness and transparency of reporting of diagnostic accuracy studies. Authors can use the list to write informative study reports. Editors and peer-reviewers can use it to evaluate whether the information has been included in manuscripts submitted for publication.

EXPLANATION

A **diagnostic accuracy study** evaluates the ability of one or more medical tests to correctly classify study participants as having a **target condition.** This can be a disease, a disease stage, response or benefit from therapy, or an event or condition in the future. A medical test can be an imaging procedure, a laboratory test, elements from history and physical examination, a combination of these, or any other method for collecting information about the current health status of a patient.

The test whose accuracy is evaluated is called **index test.** A study can evaluate the accuracy of one or more index tests. Evaluating the ability of a medical test to correctly classify patients is typically done by comparing the distribution of the index test results with those of the **reference standard**. The reference standard is the best available method for establishing the presence or absence of the target condition. An accuracy study can rely on one or more reference standards.

If test results are categorized as either positive or negative, the cross tabulation of the index test results against those of the reference standard can be used to estimate the **sensitivity** of the index test (the proportion of participants *with* the target condition who have a positive index test), and its **specificity** (the proportion *without* the target condition who have a negative index test). From this cross tabulation (sometimes referred to as the contingency or “2x2” table), several other accuracy statistics can be estimated, such as the positive and negative **predictive values** of the test. Confidence intervals around estimates of accuracy can then be calculated to quantify the statistical **precision** of the measurements.

If the index test results can take more than two values, categorization of test results as positive or negative requires a **test positivity cut-off**. When multiple such cut-offs can be defined, authors can report a receiver operating characteristic (ROC) curve which graphically represents the combination of sensitivity and specificity for each possible test positivity cut-off. The **area under the ROC curve** informs in a single numerical value about the overall diagnostic accuracy of the index test.

The **intended use** of a medical test can be diagnosis, screening, staging, monitoring, surveillance, prediction or prognosis. The **clinical role** of a test explains its position relative to existing tests in the clinical pathway. A replacement test, for example, replaces an existing test. A triage test is used before an existing test; an add-on test is used after an existing test.

Besides diagnostic accuracy, several other outcomes and statistics may be relevant in the evaluation of medical tests. Medical tests can also be used to classify patients for purposes other than diagnosis, such as staging or prognosis. The STARD list was not explicitly developed for these other outcomes, statistics, and study types, although most STARD items would still apply.

DEVELOPMENT

This STARD list was released in 2015. The 30 items were identified by an international expert group of methodologists, researchers, and editors. The guiding principle in the development of STARD was to select items that, when reported, would help readers to judge the potential for bias in the study, to appraise the applicability of the study findings and the validity of conclusions and recommendations. The list represents an update of the first version, which was published in 2003.

More information can be found on http://www.equator-network.org/reporting-guidelines/stard.

CC BY SA

For peer review only - http://bmjopen.bmj.com/site/about/guidelines.xhtml
